# Activity-based profiling of cullin–RING E3 networks by conformation-specific probes

**DOI:** 10.1038/s41589-023-01392-5

**Published:** 2023-08-31

**Authors:** Lukas T. Henneberg, Jaspal Singh, David M. Duda, Kheewoong Baek, David Yanishevski, Peter J. Murray, Matthias Mann, Sachdev S. Sidhu, Brenda A. Schulman

**Affiliations:** 1 Department of Molecular Machines and Signaling, Max Planck Institute of Biochemistry, Martinsried, Germany; 2 School of Pharmacy, University of Waterloo, Waterloo, Ontario Canada; 3 Department of Structural Biology, St. Jude Children’s Research Hospital, Memphis, TN USA; 4 Immunoregulation, Max Planck Institute of Biochemistry, Martinsried, Germany; 5 Department of Proteomics and Signal Transduction, Max Planck Institute of Biochemistry, Martinsried, Germany; 6 NNF Center for Protein Research, Faculty of Health and Medical Sciences, University of Copenhagen, Copenhagen, Denmark; 7 Present Address: Siduma Therapeutics, New Haven, CT USA

**Keywords:** Chemical modification, Post-translational modifications, X-ray crystallography

## Abstract

The cullin–RING ubiquitin ligase (CRL) network comprises over 300 unique complexes that switch from inactive to activated conformations upon site-specific cullin modification by the ubiquitin-like protein NEDD8. Assessing cellular repertoires of activated CRL complexes is critical for understanding eukaryotic regulation. However, probes surveying networks controlled by site-specific ubiquitin-like protein modifications are lacking. We developed a synthetic antibody recognizing the active conformation of NEDD8-linked cullins. Implementing the probe to profile cellular networks of activated CUL1-, CUL2-, CUL3- and CUL4-containing E3s revealed the complexes responding to stimuli. Profiling several cell types showed their baseline neddylated CRL repertoires vary, and prime efficiency of targeted protein degradation. Our probe also unveiled differential rewiring of CRL networks across distinct primary cell activation pathways. Thus, conformation-specific probes can permit nonenzymatic activity-based profiling across a system of numerous multiprotein complexes, which in the case of neddylated CRLs reveals widespread regulation and could facilitate the development of degrader drugs.

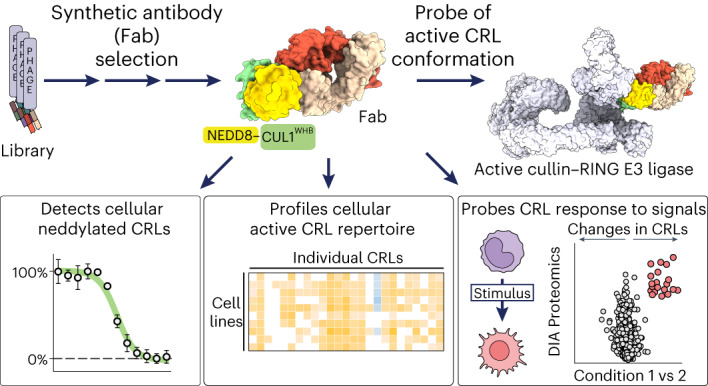

## Abstract (web summary)

Henneberg et al. developed conformation-specific antibodies enabling probing NEDD8-activated cullin–RING ubiquitin E3 ligase networks in response to extracellular stimuli, metabolic signals and degrader drugs.

## Main

Eukaryotic biology depends on widespread ubiquitylation by E3 ligases. Activities of many E3s, including those in the HECT, RBR, RCR and RZ-finger families, can be surveyed with probes reacting with the catalytic cysteine^1,2^. However, other E3s, such as the large family of cullin–RING ubiquitin ligases (CRLs), lack an active site and instead bridge substrates and ubiquitin-carrying enzymes (UCEs). Thus, alternatives to catalytic cysteine-reactive probes are needed to selectively target the active pool of CRLs.

CRLs are architecturally related complexes assembled around cores consisting of a cullin protein paired with a RING domain containing RBX protein (in humans, CUL1, CUL2, CUL3 or CUL4 with RBX1, and CUL5 with RBX2)^3–5^. CRL complexes form when a cullin’s N-terminal domain binds one of its numerous, dedicated, interchangeable substrate-binding modules (SBMs)^6^. Ultimately, the cullin’s C-terminal region and RBX1 partner with a UCE that harbors a catalytic cysteine transferring ubiquitin to the SBM-bound substrate. The modular CRL architecture, diversity of UCE partners and vast number of SBMs combinatorially generate a family of hundreds of unique E3 ligase complexes with distinct functions.

**Fig. 1:**
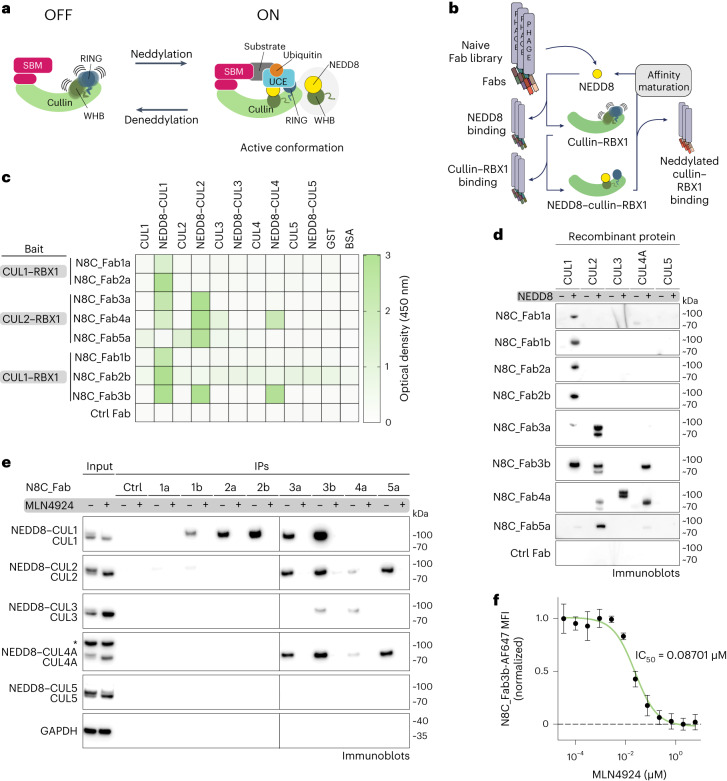
A suite of synthetic antibody fragments (Fabs) specific for NEDD8-modified CRLs. **a**, CRLs are switched ON by site-specific NEDD8 linkage to the cullin’s WHB domain. Neddylation promotes the active conformation required for ubiquitylation to be attained (SBM and UCE). **b**, Strategy to select Fabs specifically binding to NEDD8-modified, and not unmodified, CRLs. Selections were performed with neddylated C-terminal regions of CUL1 or CUL2 bound to RBX1. **c**, Binding specificity of the selected Fabs toward non-neddylated and neddylated CUL1–CUL5, GST and BSA as determined by ELISA at Fab concentrations of 50 nM (full titration in Extended Data Fig. 2c). Baits used for Fab selections are indicated. **d**, Immunoblots using indicated Fabs as primary binders for recognition of indicated purified recombinant cullins modified by NEDD8 (+) or not (−). **e**, The indicated Fabs were used in IPs from K562 cells treated with DMSO or the neddylation inhibitor MLN4924, followed by immunoblotting against cullins 1–5. Slower migrating forms of cullins lost upon MLN4924 treatment are interpreted as NEDD8-modified, whereas faster-migrating forms of cullins accumulated upon MLN4924 treatment are interpreted as unneddylated. An asterisk indicates band cross-reacting with anti-CUL4 antibody. GAPDH serves as a sample processing control. All immunoblot results (**d** and **e**) are representative of two independent experiments. **f**, MLN4924 dose–response for K562 cells measured by flow cytometry using N8C_Fab3b fluorescently labeled with Alexa Fluor 647 as a direct readout of cullin neddylation levels (*n* = 3 biologically independent samples, data are shown as mean values ± s.d.). Source data

Cellular homeostasis depends on tight regulation of CRL activity. A CRL’s ubiquitin ligase function is switched on by NEDD8 linkage to a specific site conserved across cullin C-terminal WHB subdomains^7–12^ (Fig. 1a). Studies using CUL1 have shown that modification with NEDD8 leads to a 1,000-fold increase in ubiquitylation efficiency, achieved by NEDD8 and CUL1’s WHB subdomain together adopting a specific conformation that binds and activates UCEs^11,12^. WHB domains from CUL1, CUL2, CUL3 and CUL4 are homologous, suggesting they form structurally similar complexes with covalently linked NEDD8 (refs. ^13,14^). Indeed, mutational data confirmed the importance of the NEDD8–CUL4 interface in degrader drug-induced ubiquitylation^11^.

As neddylation determines which CRLs are active, this is tightly controlled^15^. Specialized E1-E2-E3 cascades catalyze NEDD8 linkage to cullins. Meanwhile, the COP9 signalosome (CSN) deconjugates NEDD8 unless a CRL is shielded by binding a substrate^16–21^. The current model is that after substrate degradation, most CRLs are deneddylated. Studies of CUL1- and CUL4-based CRLs indicated that only when deneddylated, these CRLs and presumably others are subject to a pathway promoting SBM dissociation from their cullin–RING partners^22–25^. Some CRLs are subject to additional controls in the absence of substrate, including SBM autoubiquitylation and/or formation of autoinhibited self-assemblies^21,26–28^. Thus, cellular CRL repertoires are dynamically reshaped through assembly, activation, deactivation and disassembly. As such, NEDD8 linkage to a cullin is typically a marker of an assembled, active CRL^5,29^.

Regulation of the CRL network by neddylation has been implicated in orchestrating cell division, immune signaling, DNA replication and repair, responses to redox stress and hypoxia, tumorigenesis and hijacking by bacterial and viral pathogens^4,6,30^. Neddylation is also important for CRL-dependent targeted protein degradation^21,31,32^. Furthermore, surveying the effect of inhibiting neddylation on CUL1- and CUL4-associated SBMs led to an ‘adaptive exchange hypothesis’ proposing that the landscape of NEDD8-activated CRLs is rewired to adapt to changes in cellular conditions^22–24^. Thus, it is of great interest to probe NEDD8-activated CRLs. However, the current method of assessing active CRL repertoires requires endogenous cullin tagging^22,24^. Endogenous tagging is laborious, may introduce artifacts, limits studies to the engineered cell line and can be challenging for primary cells. Targeting NEDD8 is likewise complicated as a major population of NEDD8 in cells is unconjugated^33,34^. The use of anti-NEDD8 antibodies could be problematic given that much of NEDD8’s surface is buried by interactions with a cullin^11,12,35^. Furthermore, previous studies using tagged NEDD8 identified only a small subset of SBMs in affinity purification mass spectrometry (AP–MS) experiments, substantially fewer compared to the same workflow applied to identically tagged cullins^36,37^. Moreover, several hundred proteins in addition to cullins undergo neddylation^33,34^.

To address these challenges, we took inspiration from the successful targeting of ubiquitin chains with affinity reagents^38–41^ and used phage display to generate antigen-binding fragments (Fabs) that selectivity target neddylated cullins with nanomolar affinities. Structural studies show one of the Fabs binds neddylated CUL1 in the active conformation during ubiquitylation. Biochemistry and proteomics reveal it not only recognizes NEDD8-linked cullin proteins but also captures neddylated CRL1, CRL2, CRL3 and CRL4 complexes with high specificity. Combining this activity-based probe with quantitative proteomics allows the profiling of distinct active CRL complex landscapes and their responses to cellular signaling pathways and degrader drugs.

## Results

### A suite of synthetic antibodies recognizing neddylated CRLs

**Extended Data Fig. 1:**
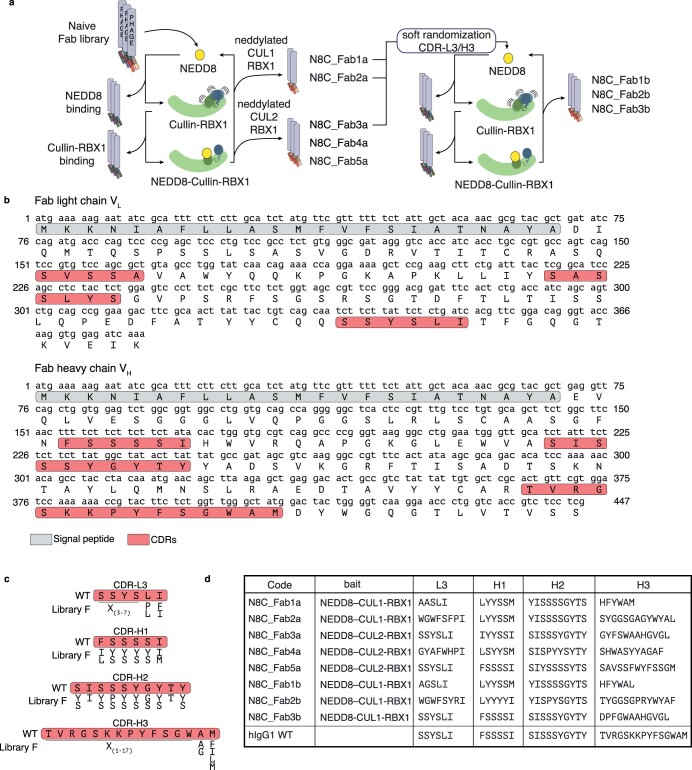
Selection of Fabs targeting neddylated cullins. **a**, Schematic representation of the negative>negative>positive selection strategy used to generate synthetic antibodies (Fabs) targeting neddylated but not unneddylated cullins indicating steps in the process specific Fabs were produced. Selections were performed with neddylated C-terminal regions of CUL1 or CUL2 bound to RBX1. **b**, DNA and amino acid sequences of the library F Fab scaffold with CDRs highlighted in red. **c**, Overview of CDR diversity of library F with allowed amino acids for each position indicated. For the position labeled with X, amino acids Tyr, Ser, Gly, Ala, Phe, Trp, His, Pro or Val were permitted. **d**, Sequence information of Fabs generated with selection strategy in (a) and the baits used for their selection.

To generate probes selectively binding neddylated CRLs, we established a negative>negative>positive selection strategy enriching specific binders from a library of Fabs on phage^42^. First, the library was depleted of Fabs recognizing a cullin–RING complex or NEDD8 without the other. Next, a neddylated cullin–RING complex was the bait for positive selection (Fig. 1b and Extended Data Fig. 1a,b). The baits were minimal complexes between RBX1 and the C-terminal regions of cullins that can be enzymatically neddylated^7^. Performing independent selections with CUL1 or CUL2 yielded two and three Fab sequences, respectively.

**Extended Data Fig. 2:**
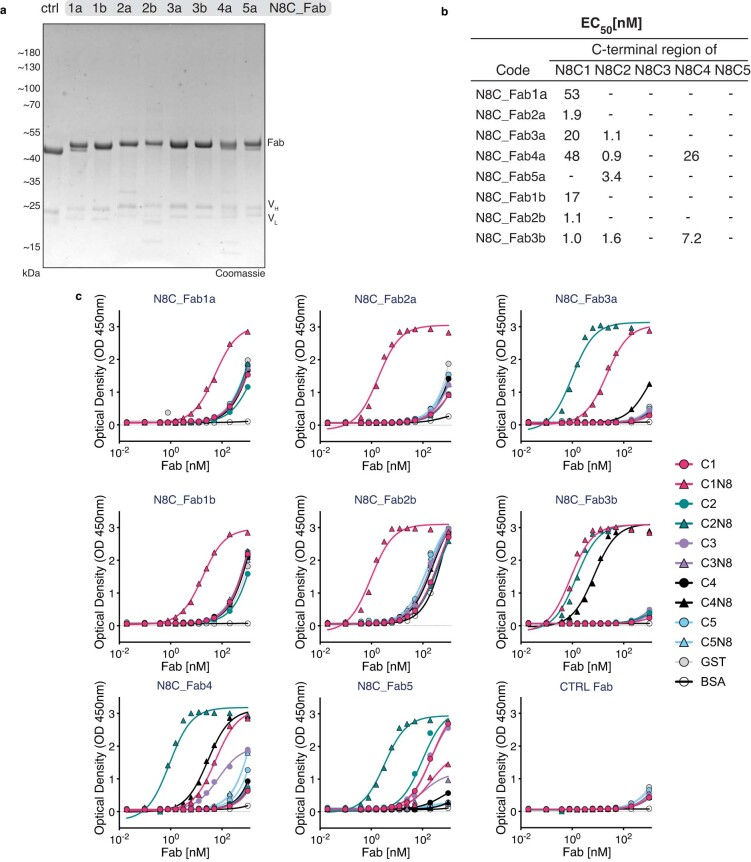
Characterization of Fabs targeting neddylated cullins. **a**, Coomassie stained gel of indicated Fabs from the N8C_Fab suite. Shown results are representative of two independent experiments. **b**, EC_50_ values for binding between indicated Fabs and C-terminal regions of neddylated cullins 1-5 (N8 C1 = NEDD8-modified CUL1, and so on) as determined by ELISA. **c**, ELISA curves of Fab binding titrations against neddylated and non-neddylated versions of cullins 1-5, GST and BSA to determine binding specificities shown in Fig. 1c and EC_50_ values shown above in panel b. Source data

Affinities and specificities of the selected phage-displayed Fabs were assessed by enzyme-linked immunosorbent assays (ELISAs; Extended Data Fig. 2a–c). The Fabs specifically bound neddylated cullins, and exhibited little to no binding to unneddylated cullins, GST or BSA (Fig. 1c). Panning across the cullin family showed several Fabs were specific for the cullins used as baits in their selection. Unexpectedly, two Fabs selected to bind a neddylated version of CUL2–RBX1 displayed broader interactions, with neddylated CUL1–RBX1, and one also with neddylated CUL4A–RBX1.

**Extended Data Fig. 3:**
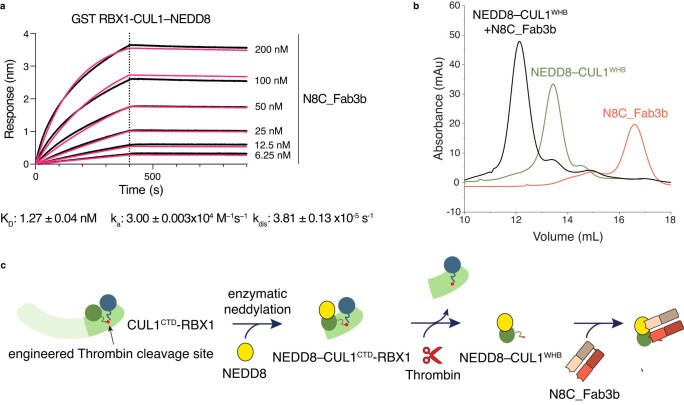
Crystallization of the neddylated CUL1 WHB domain in complex with N8C_Fab3b. **a**, Biolayer Interferometry sensorgram (black) and curve fitting (red) of N8C_Fab3b (analyte) binding to GST RBX1-CUL1–NEDD8 (ligand, using GST biosensors). Calculated values for equilibrium constant (K_D_) and rates of association and dissociation (k_a_ and k_dis_) are shown below. **b**, Size exclusion chromatograms comparing migrations of N8C_Fab3b, NEDD8–CUL1^WHB^, and the complex of the two as visualized by total absorbance (280 nm). The purified complex was crystallized for structure determination. **c**, Overview of the strategy employed to generate the isolated neddylated CUL1 WHB subdomain. A mutant version of the complex between RBX1 and CUL1’s C-terminal region (CTD) was engineered with a thrombin cleavage site upstream of CUL1’s WHB subdomain. After enzymatic neddylation of RBX1 bound to CUL1, thrombin cleavage released the NEDD8–CUL1^WHB^ unit, which was mixed with N8C_Fab3b. Source data

To improve affinities for CUL1, and to investigate if an orthogonal selection could extend the range of neddylated cullins recognized by a single Fab, we performed another round of selections using the neddylated CUL1 fragment bound to RBX1 as the bait. New libraries were based on the sequences of N8C_Fab1a, N8C_Fab2a and N8C_Fab3a, with soft randomization in their complementarity-determining regions (CDRs)-L3 and H3. Selections with the libraries based on N8C_Fab1a and 2a yielded new Fabs with up to threefold increased affinity compared to their original counterparts (Extended Data Fig. 2b). The selection with the library based on the framework of N8C_Fab3a led to N8C_Fab3b, with the following remarkable properties by ELISA: maintaining interaction with neddylated CUL2–RBX1, 20-fold improvement in EC_50_ toward neddylated CUL1–RBX1 and emergent recognition of neddylated CUL4A–RBX1. Furthermore, N8C_Fab3b copurified with its bait in size-exclusion chromatography (SEC). Bio-Layer Interferometry measurements indicated a nanomolar affinity for neddylated CUL1–RBX1, with association and dissociation rates of ~3 × 10^4^ M^−1^ s^−1^ and ~3.8 × 10^−5^ s^−1^, respectively (Extended Data Fig. 3a,b).

Overall, the selections yielded eight Fabs. Some specifically bind neddylated versions of either CUL1–RBX1 or CUL2–RBX1. Others recognize multiple neddylated cullin–RBX1 complexes with low nanomolar EC_50_s.

### N8C_Fabs selectively detect neddylated cullins

We tested purified versions of the Fabs for selective detection of neddylated cullins in various assays. In immunoblots, they all specifically recognized neddylated cullins. Here cullin preferences correlated with those of the phage-displayed Fabs detected by ELISA (Fig. 1d). The trends also held when the Fabs were used to perform immunoprecipitation (IP) from K562 cell lysates, followed by immunoblotting with commercial cullin-specific antibodies. Neddylation dependence was confirmed by cell treatment with the neddylation inhibitor MLN4924 eliminating interactions^29^ (Fig. 1e). Although neddylated CUL3–RBX1 was not detected by ELISA as interacting with any of the Fabs when displayed on phage, it was detected by purified N8C_Fab4a in immunoblot and enriched by IP with both N8C_Fab3b and N8C_Fab4a from cell lysates.

Based on its high specificity for neddylated over unneddylated cullins, and capacity to bind multiple cullins, we tested N8C_Fab3b for utility in flow cytometry. If the N8C_Fab3b directly detected neddylated cullins, then signal would be eliminated by treatment with MLN4924. Indeed, dose–response curves for K562 cells showed a half-maximal inhibitory concentration (IC_50_) of ~87 nM (Fig. 1f), in line with the <100 nM reported based on NEDD8 migration detected in immunoblots as a proxy for conjugate formation^29^.

### Effects of N8C_Fabs on neddylated CRL activities

**Fig. 2:**
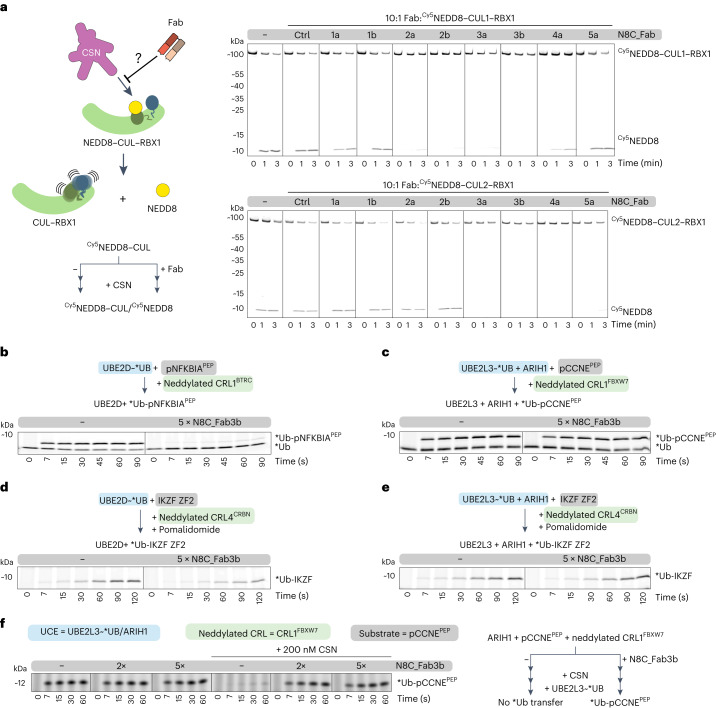
Effects of neddylated cullin targeting Fabs on CRL activities. **a**, CSN-catalyzed NEDD8 deconjugation is visualized by loss of fluorescent NEDD8 signal from complex with CUL1 and accumulation of free NEDD8 observed over time by SDS–PAGE. Assays test protective effects of incubating 10× molar excess of indicated Fabs with neddylated C-terminal regions of CUL1 or CUL2 complexes with RBX1. **b**, Effects of N8C_Fab3b binding on ubiquitylation by neddylated CRL1^BTRC^-UBE2D as determined by monitoring transfer of fluorescent ubiquitin (*Ub) to a substrate peptide derived from phospho-NFKBIA (pNFKBIA^PEP^) by SDS–PAGE. **c**, Same as **b** but monitoring the transfer of *Ub by neddylated CRL1^FBXW7^-UBE2L3/ARIH1 to substrate peptide derived from phospho-Cyclin E (pCCNE^PEP^). **d**, Same as **b** but monitoring pomalidomide-induced transfer of *Ub by neddylated CRL4^CRBN^-UBE2D to the IKZF ZF2 substrate. **e**, Same as **b** but monitoring pomalidomide-induced transfer of *Ub by neddylated CRL4^CRBN^-UBE2L3/ARIH1 to the IKZF ZF2 substrate. **f**, Same as **c** but comparing *Ub transfer in the absence and presence of CSN with or without prior incubation with N8C_Fab3b. For schemes of reactions in **b**–**f**, the UCE is in highlighted blue, the substrate is in gray and the neddylated cullin is in green. Gel panels (**a**–**f**) are representative of two independent experiments. Source data

Neddylation alters the binding partners and functions of cullin–RING complexes. Thus, we tested the effects of adding N8C_Fabs to activity assays. First, we explored whether a Fab could protect the fragile NEDD8 mark from CSN-mediated deconjugation. Whereas CSN rapidly catalyzed NEDD8 removal from CUL1–RBX1 and CUL2–RBX1 complexes, the addition of several of the N8C_Fabs to these reactions slowed deneddylation (Fig. 2a). Retention of the NEDD8 linkage largely correlated with Fab binding measured by ELISA (Extended Data Fig. 2b), with some exceptions. For example, N8C_Fab4a provided robust protection of neddylated CUL1 compared to N8C_Fab1b despite almost threefold higher EC_50_. A possible explanation for the differences would be if only a subset of the Fabs binds in such a way as to prevent CSN access to the NEDD8–cullin bond.

We selected N8C_Fab3b for further characterization based on its binding the broadest range of neddylated cullins, because it maintained cullin neddylation in the presence of CSN. We tested the effects on structurally characterized ubiquitylation reactions. The UCEs were either an E2 (UBE2D)^11^ or E3 (ARIH1, which collaborates with the E2 UBE2L3 to ubiquitylate CRL substrates)^10,12^. N8C_Fab3b inhibited UBE2D- and neddylated CRL1^BTRC^-dependent ubiquitylation of a peptide substrate derived from phospho-NFKBIA (Fig. 2b). Meanwhile, adding N8C_Fab3b did not affect UBE2L3/ARIH1- and neddylated CRL1^FBXW7^-mediated ubiquitylation of a peptide substrate derived from phospho-Cyclin E (Fig. 2c). The distinct effects of N8C_Fab3b correlated with the UCE used in the reaction, as shown by examining pomalidomide-induced CRL4^CRBN^-mediated ubiquitylation of a peptide substrate based on the Ikaros degron (Fig. 2d,e).

The lack of effects of N8C_Fab3b on reactions with ARIH1 could be explained in either of two ways. N8C_Fab3b might not bind during ARIH1-dependent ubiquitylation. Alternatively, N8C_Fab3b might be compatible with ubiquitylation by ARIH1. We devised an experiment distinguishing the possibilities, based on competition between N8C_Fab3b and deneddylation, and requirement for CRL neddylation for ARIH1-mediated ubiquitylation (Fig. 2f). The addition of CSN at a concentration overcoming CRL substrate inhibition eliminated UBE2L3/ARIH1-mediated ubiquitylation. However, ubiquitylation activity was restored by N8C_Fab3b. Thus, N8C_Fab3b protects the neddylated CRL1 complex during ARIH1-mediated ubiquitylation.

### N8C_Fab3b captures the active conformation of NEDD8–CUL1

To understand the molecular basis for selective recognition, we sought the structure of an N8C_Fab3b complex with a neddylated cullin. We devised a strategy to isolate enzymatically neddylated CUL1 WHB domain (Extended Data Fig. 3c), which cocrystallized with N8C_Fab3b, yielding a structure at 2.7 Å resolution (Supplementary Table 1).

**Fig. 3:**
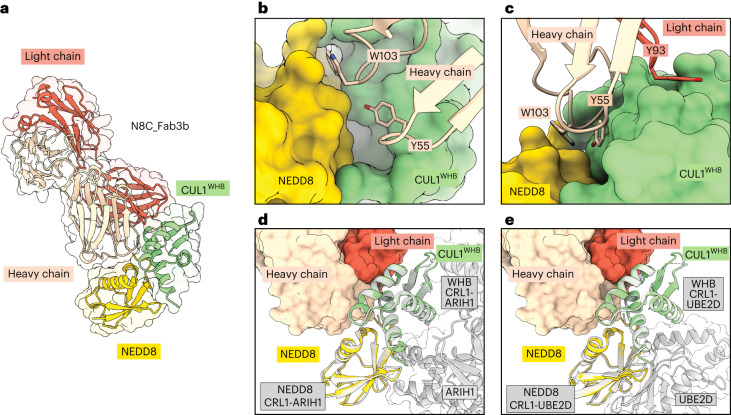
Crystal structure shows N8C_Fab3b binds active conformation of neddylated CUL1 WHB domain. **a**, Crystal structure of N8C_Fab3b in complex with neddylated CUL1 WHB domain (CUL1^WHB^). N8C_Fab3b recognizes a unique interface spanning both NEDD8 and CUL1. **b**, Closeup of the N8C_Fab3b heavy chain Y55 and W103 buried in groove between the CUL1 WHB domain and NEDD8. **c**, Closeup showing N8C_Fab3b light chain Y93 hooking into the edge of the CUL1 WHB domain. **d**, Superposition over the NEDD8-modified CUL1 WHB domain from structure of N8C_Fab3b-bound complex and in an active CRL1^FBXW7^-UBE2L3/ARIH1 complex (7B5L). N8C_Fab3b captures the active conformation of NEDD8 and its covalently linked CUL1 WHB domain. **e**, Superposition over the NEDD8-modified CUL1 WHB domain from structure of N8C_Fab3b-bound complex and in the active CRL1^BTRC^-UBE2D2 complex (6TTU).

**Extended Data Fig. 4:**
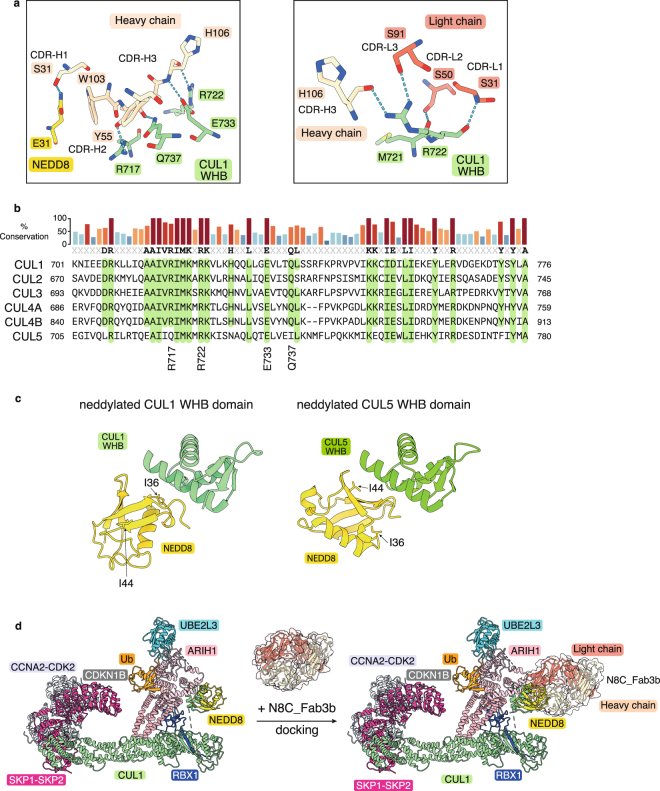
Structure of N8C_Fab3b in complex with the neddylated CUL1 WHB domain. **a**, Detailed views of the interactions between light and heavy chain of N8C_Fab3b and the CUL1 WHB domain and NEDD8. **b**, Sequence alignment of the WHB domains of cullins 1-5 with conserved residues highlighted in green and key residues involved in the interaction with N8C_Fab3b indicated below. Bars indicate percentage of sequence conservation. **c**, Structural comparison of the arrangement between NEDD8 and its linked WHB domain from CUL1 (structure shown in Fig. 3) or CUL5 (PDB: 7ONI). **d**, Modeling N8C_Fab3b on the structure of the neddylated CRL1^SKP2^-UBE2L3/ARIH1 complex (PDB: 7B5L), aligned over NEDD8-linked to the CUL1 WHB domain from both structures.

The structure reveals the strict requirement for cullin neddylation as follows: N8C_Fab3b binds a unique interface spanning both NEDD8 and CUL1 (Fig. 3a). Tyr55 and Trp103 of N8C_Fab3b CDR-H2 and CDR-H3, respectively, insert into a groove between NEDD8’s so-called Ile36 patch and the CUL1 WHB domain. One edge of this groove is the isopeptide linkage between NEDD8 and CUL1, and the other is established by noncovalent NEDD8–CUL1 contacts (Fig. 3b). The complex is stabilized by multiple hydrogen bonds between the Fab CDRs and either NEDD8 or CUL1 (Extended Data Fig. 4a), as well as Tyr93 of CDR-L3 clasping the edge of CUL1’s WHB domain (Fig. 3c). As such, N8C_Fab3b binds a specific arrangement of NEDD8 and its linked CUL1 WHB domain.

Remarkably, the arrangement of the N8C_Fab3b-bound NEDD8–CUL1 WHB unit matches that in structures representing neddylated CRL1^BTRC^ ubiquitylation with the E2 UBE2D^11^ and neddylated CRL1^SKP2^ and CRL1^FBXW7^ ubiquitylation with the E2/E3 combination UBE2L3/ARIH1 (ref. ^12^; Fig. 3d,e). The key WHB domain residues binding noncovalently to NEDD8 are conserved in cullins 1–4 (Extended Data Fig. 4b). However, CUL5’s sequence is incompatible with forming such a complex; NEDD8 and CUL5’s WHB domain adopt a different conformation in neddylated CRL5 E3s^35^ (Extended Data Fig. 4c). Thus, selectivity is determined both by the interactions mediated directly by N8C_Fab3b and also by the capacity for a neddylated CRL to form the active arrangement between NEDD8 and a cullin’s WHB domain.

NEDD8 and CUL1’s WHB domain are thought to sample multiple conformations in a neddylated CRL1 complex; they were not visualized in previous cryo-EM structures without a UCE, and the NEDD8–CUL1 WHB domain unit occupies different relative positions to activate UBE2D or ARIH1 (refs. ^11,12^). Docking N8C_Fab3b onto the prior structures of ubiquitylation complexes shows that although it would clash during ubiquitin transfer from UBE2D to an SBM-bound substrate, this Fab can capture an ARIH1-bound CRL complex (Extended Data Fig. 4d). This explains effects of N8C_Fab3b on the different ubiquitylation reactions (Fig. 2b–e). The structure with N8C_Fab3b also suggests NEDD8 and a cullin’s WHB domain have an intrinsic propensity to bind each other in the active conformation. Together, the data show that N8C_Fab3b captures the active arrangement between NEDD8 and its linked cullin domain, and reveal its potential to probe for NEDD8-activated CRLs.

### A pipeline probing cellular repertoires of neddylated CRLs

**Fig. 4:**
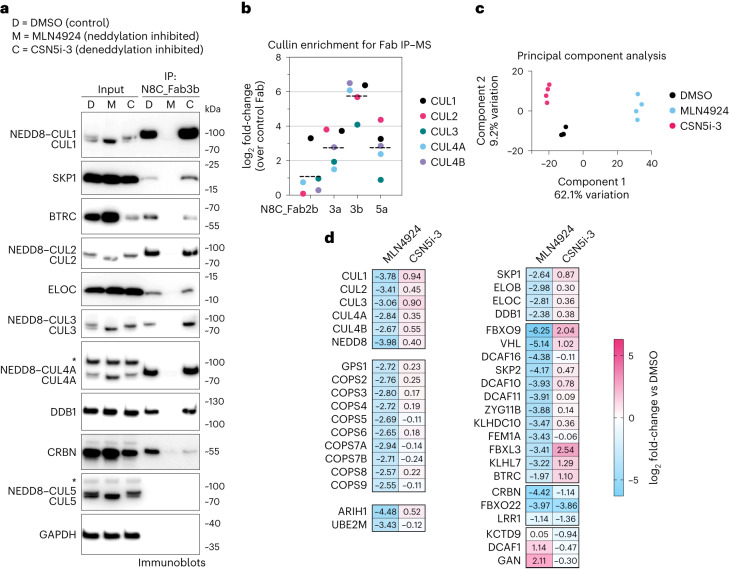
Probing the cellular repertoire of active CRLs. **a**, IPs using N8C_Fab3b from K562 cells treated with DMSO (D), the neddylation inhibitor MLN4924 (M) or the cullin deneddylase inhibitor CSN5i-3 (C) for 2 h, probed for indicated CRL components by immunoblotting. Slower migrating forms of cullins lost by MLN4924 treatment are interpreted as NEDD8-modified, whereas faster-migrating forms accumulating upon MLN4924 treatment are interpreted as unneddylated. An asterisk indicates band cross-reacting with anti-CUL4 or anti-CUL5 antibody. Shown results are representative of two independent experiments. GAPDH serves as a sample processing control. **b**, Comparison of cullins detected by MS in IPs with Fabs N8C_Fab2b, N8C_Fab3a, N8C_Fab3b and N8C_Fab5a compared to a control Fab (*n* = 3 biological independent samples). Mean log_2_(fold change) is indicated by a dashed line. **c**, PCA plot of protein groups identified by MS of N8C_Fab3b IPs from 293 T cells treated with DMSO (black), MLN4924 (blue) or CSN5i-3 (magenta) for 2 h. Each dot corresponds to a biological replicate (*n* = 3). **d**, Heatmap of selected proteins differentially identified by MS from N8C_Fab3b IPs from 293 T cells either treated with MLN4924 compared to DMSO control or treated with CSN5i-3 compared to DMSO control (*n* = 3). Source data

**Extended Data Fig. 5:**
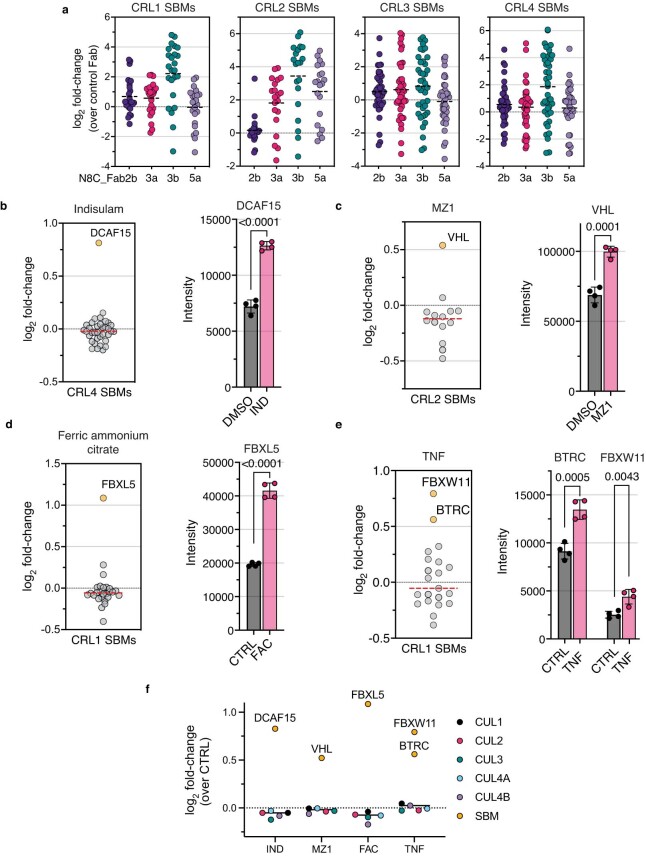
IP-MS experiments using N8C_Fab3b. **a**, Comparison of CRL1, CRL2, CRL3 and CRL4 SBMs enriched in IP-MS experiments with Fabs N8C_Fab2b, 3a, 3b, and 5a as compared to a control Fab from the same data used for Fig. 4b (n = 3 biological independent samples). Log_2_ fold-difference is shown. Each dot represents one SBM known to associate with the indicated cullin. **b-f**, Further analysis of data shown in Fig. 5 (n = 4 biological independent samples, data are shown as mean +/- SD). **b**, Reshaping of the CRL network by molecular glue degrader molecule, specifically 1 hour treatment of 293T cells with 2 µM Indisulam (IND) compared to control (DMSO) as determined by SBMs identified in N8C_Fab3b IP-MS. On left, each dot represents one CRL4 SBM and the log_2_ fold-difference between treated samples and the control (DMSO) is shown. On right is bar graph showing differences in protein group intensity for indicated SBM with the p-value determined by two-sided Student’s t-test. **c**, Reshaping of the CRL network by bivalent degrader molecule, specifically 1 hour treatment of 293T cells with 1 µM MZ1 compared to DMSO control as determined by SBMs identified in N8C_Fab3b IP-MS. On left, each dot represents one CRL2 SBM and the log_2_ fold-difference between treated samples and the control (DMSO) is shown. On right is bar graph showing differences in protein group intensity for indicated SBM with the p-value determined by Student’s t-test. **d**, Reshaping of the CRL network after metabolic signal, specifically 90 minute treatment of 293T cells with 100 µM ferric ammonium citrate (FAC) compared to treatment with PBS as the control as determined by SBMs identified in N8C_Fab3b IP-MS. On left, each dot represents one CRL1 SBM and the log_2_ fold-difference between treated samples and the control (PBS) is shown. On right is bar graph showing differences in protein group intensity for indicated SBM with the p-value determined by Student’s t-test. **e**, Reshaping of the CRL network after cytokine signal, specifically treatment of K562 cells with 25 ng/ml TNF for 5 min compared to PBS control as determined by SBMs identified in N8C_Fab3b IP-MS. On left, each dot represents one CRL1 SBM and the log_2_ fold-difference between treated samples and the control (PBS) is shown. On right is bar graph showing differences in protein group intensity for indicated SBMs with the p-values determined by Student’s t-test. **f**, Analysis of experiments in panels b-e, showing log_2_ fold-differences for each cullin as a dot (CUL1 in black, CUL2 in magenta, CUL3 in green, CUL4A in blue, CUL4B in purple) SBM of interest in yellow. The cellular signal is indicated below. Source data

Given that N8C_Fab3b IPs could enrich NEDD8-activated CRLs, we next optimized conditions to establish a pipeline meeting several key criteria. First, we sought to identify proteins interacting specifically with neddylated cullins. This was distinguished by comparing the effects of treating cells with DMSO as control or MLN4924 to eliminate cullin neddylation^29^. We also examined the effects of disrupting regulation by treating cells with the CSN inhibitor CSN5i-3 (ref. ^43^). Second, an elaborate CRL assembly and disassembly pathway shuffles the limited cellular pool of cullin–RBX1 subcomplexes between excess SBMs in a deneddylation-dependent process^22,24,25^. CRL disassembly is transiently paused by the retention of NEDD8 on the complexes bound to substrates. To preserve the cellular repertoire of neddylated CRLs, postlysis cullin reshuffling must be prevented by an ‘N8-block’, where MLN4924 and CSN5i-3 are applied during cell harvesting, and included in lysis and wash buffers^24^. Performing IPs with N8C_Fab3b using an N8-block, and immunoblotting indeed showed neddylation-dependent enrichment of CRL components such as adapter proteins SKP1, ELOC and DDB1, and SBMs including BTRC and CRBN (Fig. 4a). To confirm N8C_Fab3b is most suited for profiling active CRL interactors, we performed IPs with N8C_Fab2b, N8C_Fab3a and N8C_Fab5a, followed by library-free data-independent acquisition (DIA) MS. The Fabs all substantially enriched cullins and SBMs in a neddylation-dependent manner (Fig. 4b and Extended Data Fig. 5a). Their cullin specificities largely paralleled in vitro binding properties (Extended Data Fig. 1c). N8C_Fab3b on average yielded tenfold greater enrichment of known cullin-associated proteins compared to the next best Fab, so we selected it to profile cellular activated CRL-omes (Fig. 4b).

We next used unbiased proteomics in DIA format to probe the interactome of N8C_Fab3b. Principal component analysis of IPs from 293T cells treated with DMSO, MLN4924 or CSN5i-3 highlighted reproducibility and neddylation dependence across four biological replicates (Fig. 4c). The analysis readily identified interactors known to vary across the differing neddylation states imposed by the inhibitors, providing positive controls (Fig. 4d). Besides the cullins and NEDD8, the interactome included the components of the COP9 signalosome, the RBX1-specific NEDD8 E2 UBE2M and UCE ARIH1 as well as adapter proteins SKP1, ELOB, ELOC and DDB1, all of which showed strong neddylation dependence. Notably, the CUL5–RBX2-specific NEDD8 E2 UBE2F and UCE ARIH2 were not observed, indicating that the interactors are with neddylated cullins 1–4 recognized by N8C_Fab3b.

SBM interactions are sectored into three categories with respect to inhibiting neddylation with MLN4924 or deneddylation with CSN5-i3 (Fig. 4d). The majority decrease with MLN4924, and either remain similarly bound or increase with CSN5i-3 treatments, consistent with the model for regulation by neddylation, deneddylation, assembly and disassembly^22–25^. Other SBMs decrease upon inhibiting either neddylation or deneddylation. This behavior has been reported previously, and would be explained by autoubiquitylation-mediated degradation as demonstrated for CRBN^21,27^. Finally, KCTD9, DCAF1 and GAN increased in the IPs after MLN4924 treatment. Such preferential association with neddylated cullins in the absence of ongoing neddylation suggests that these SBMs have unconventional means to block CSN. This property, coincident with SBM autoubiquitylation, has been recently reported for a self-assembly formed by another neddylated CRL subject to pleiotropic regulation^28^. Notably, KCTD9, DCAF1 and GAN all form higher-order assemblies, and unneddylated CRL4^DCAF1^ oligomerizes and sequesters its CUL4 from neddylation and CSN^26,44,45^. Thus, we speculate that this third SBM class forms specialized assemblies retaining neddylated cullins upon MLN4924 treatment, although future studies will be required to determine the precise molecular mechanisms.

### Profiling CRL complexes activated by extracellular signals

The results from Fig. 4d suggested that N8C_Fab3b could identify CRL complexes switching neddylation state in response to external stimuli. To explore this further, we tested examples of three types of stimuli known to trigger neddylated CRL-dependent protein degradation, using a protocol that inhibits protein turnover.

**Fig. 5:**
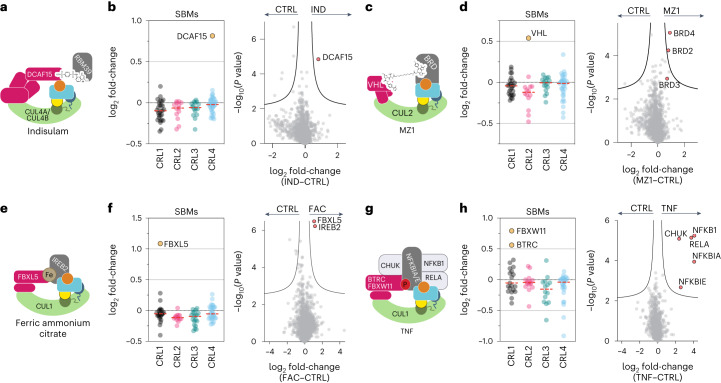
Profiling CRL complexes activated by extracellular signals. **a**, Cartoon representing the neddylated CRL4^DCAF15^ complex, wherein the degrader molecule Indisulam recruits RBM39 as a neosubstrate. NEDD8, cullin WHB domain, RBX1 RING domain, ubiquitin and UCE are colored yellow, dark green, blue, orange and cyan, respectively, as in Fig. 1a. **b**, log_2_(fold change) in SBMs for CUL1, CUL2, CUL3 and CUL4 (left) and all identified protein groups (right, volcano plot) in N8C_Fab3b IPs from 293 T cells treated for 1 h with 2 µM Indisulam (IND) versus DMSO (CTRL). Mean log_2_(fold change) is indicated by a dashed line (left). For the volcano plot, the curve for 5% FDR (FDR controlled, two-sided *t* test, randomizations = 250, s0 = 0.1) is shown. **c**, Cartoon representing the neddylated CRL2^VHL^ complex, wherein MZ1 recruits a BRD protein as a neosubstrate. **d**, Same as **b** but with different cellular treatments: 1 h with 1 µM MZ1 versus DMSO (CTRL). **e**, Cartoon representing the neddylated CRL1^FBXL5^ complex, wherein the substrate IREB2 is recruited in an iron-dependent manner. **f**, Same as **b** but with different cellular treatments: 90 min with 100 µM ferric ammonium citrate (FAC) versus PBS (CTRL). **g**, TNF stimulation causes phosphorylation of NFKBIA and NFKBIE and their subsequent ubiquitylation by CRL1^BTRC^ and CRL1^FBXW11^. The known assembly of neddylated CRL1^BTRC^ or CRL1^FBXW11^ (simplified here to monomeric SBM) with phosphorylated NFKBIA or NFKBIE is shown colored in cartoon. **h**, Same as **b** but with different cells and treatments: 5 min treatment of K562 cells with 25 ng ml^−1^ TNF versus PBS (CTRL). All experiments shown in **b**, **d**, **f** and **h** included *n* = 4 biological independent samples. Source data

Given the emerging importance of neddylated CRLs in targeted protein degradation^32,46^, we profiled responses to degrader drugs. First, we examined a molecular glue, Indisulam, which engages neddylated CRL4^DCAF15^ to degrade RBM39 (refs. ^47,48^; Fig. 5a). Indisulam was selected as a benchmark, due to its known dependence on neddylation/deneddylation and CRL assembly/disassembly machineries^21,24^. Indeed, profiling with N8C_Fab3b identified DCAF15 as increasing in neddylated cullin association upon Indisulam treatment (Extended Data Fig. 5b). Strikingly, this was the only significant change triggered by Indisulam (Fig. 5b). We next examined effects of a bivalent degrader, MZ1, that affixes complexes between CRL2^VHL^ and bromodomain and extraterminal domain (BET) family members BRD2, BRD3 and BRD4 (ref. ^49^; Fig. 5c). Our active CRL profiling showed MZ1 triggers enrichment of VHL (Extended Data Fig. 5c). It also revealed BRD2, BRD3 and BRD4 as associating with a neddylated CRL in an MZ1-dependent manner (Fig. 5d). Thus, our profiling method can identify the CRL activated by a degrader drug, and in some cases targets for degradation as well. The preferential enrichment of BRD4 over BRD2 and BRD3 correlates with MZ1-targeted degradation rather than its affinity for these neosubstrates^49^, in accordance with the concept that CRL complex architecture rather than substrate binding is the driver of degradation^46,50^.

Many endogenous cellular signaling pathways depend on CRL-based responses to execute biological functions. To determine if profiling with N8C_Fab3b permits capturing such regulation, we first examined a metabolic signaling pathway. High iron has been shown to trigger CRL1^FBXL5^-dependent degradation of iron regulatory protein 2 (IREB^51,52^ ; Fig. 5e). Indeed, treatment of cells with ferric ammonium citrate elicited ~twofold relative increases only in FBXL5 and its substrate IREB2 as neddylated cullin-associated proteins (Fig. 5f and Extended Data Fig. 5d). Finally, we also profiled the response to a cytokine. TNF induces degradation of phosphorylated NFKBIA and NFKBIE by CRL1^BTRC^ or CRL1^FBXW11^ (also called CRL1^βTRCP1^ and CRL1^βTRCP2^ (ref. ^53^); Fig. 5g). Accordingly, these SBMs and substrates were selectively identified by our active CRL probing method upon cell treatment with TNF (Fig. 5f and Extended Data Fig. 5e). Notably, as also observed upon treatment with the other stimuli, our profiling method identified the precise SBM-containing complex that responded to the signal, even though the cellular levels of neddylated cullins were generally unchanged (Extended Data Fig. 5f). Remarkably, here the profiling also identified other key components of TNF-regulated degradation and signaling pathways as follows: specifically, the kinase CHUK responsible for generating the NFKBIA and NFKBIE phospho-degrons, and the transcription factors NFKB1 and RELA (Fig. 5f). Thus, our workflow also identifies proteins involved in signaling pathways associated with ubiquitylated substrates.

### Baseline active CRL repertoire primes cellular response

**Fig. 6:**
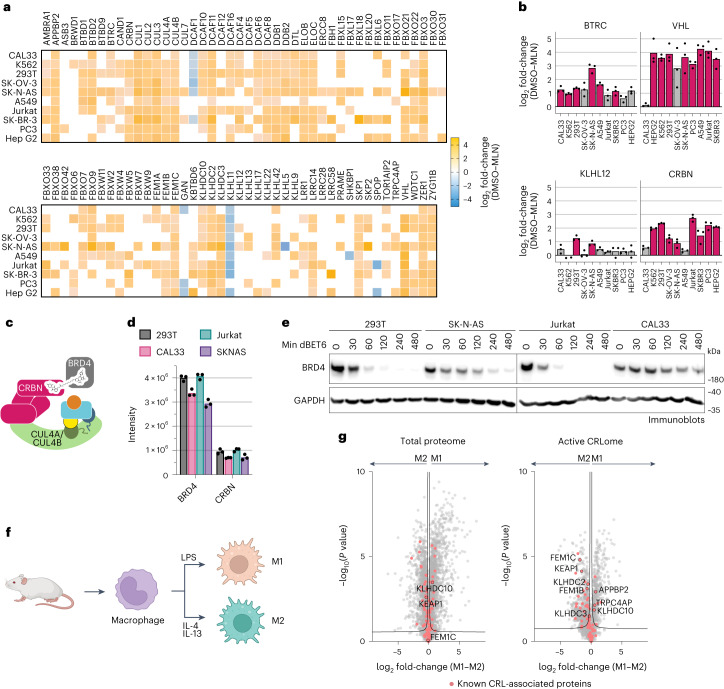
Baseline neddylated CRL repertoire primes cellular response. **a**, Heatmap representation of N8C_Fab3b-based CRL profiling in different human cell lines. log_2_(fold change) comparing DMSO control versus MLN4924-treated cells with all listed SBMs being substantially enriched over control in at least one cell line (*n* = 3 biological independent samples, FDR controlled at 5% cutoff, two-sided *t* test, randomizations = 250, s0 = 0.1). SBMs not substantially enriched over control in a particular cell line are not colored. **b**, Bar graphs based on **a** represent differences in the identified levels of selected CRL1, CRL2, CRL3 and CRL4 SBMs (BTRC, VHL, KLHL12 and CRBN, respectively) to highlight variations in CRL repertoires across cell lines. Data are shown as mean values. Pink bars indicate significant difference in N8C_Fab3b enrichment between DMSO and MLN4924 treatment. **c**, Cartoon representing the neddylated CRL4^CRBN^ complex, wherein the degrader molecule dBET6 recruits BRD4 as a neosubstrate. **d**, Bar graph of protein group intensities as determined by total proteomics as a readout of cellular protein levels for BRD4 and CRBN in 293T, CAL33, Jurkat and SKNAS (*n* = 3 biological independent samples, data are shown as mean values). **e**, Time courses of BRD4 degradation induced by treatment of 293T, SK-N-AS, Jurkat and CAL33 cells with 0.1 µM dBET6. BRD4 and GAPDH loading control were monitored by immunoblotting. Shown results are representative of two independent experiments. **f**, Schematic of production of mouse bone marrow-derived macrophages and activation to M1 and M2 states by treatment with LPS or a combination of IL-4 and IL-13, respectively. **g**, Volcano plots of the differences between M1 and M2 mouse macrophages seen in the total proteome (*n* = 4; left) and active CRLome (*n* = 4) as determined by N8C_Fab3b-based profiling (right). Known CRL components are indicated in red. SBMs associated with redox stress responses and/or recognition of substrate C-degrons are indicated with their names. Curves for 5% FDR (FDR controlled, two-sided *t* test, randomizations = 250, s0 = 0.1) are shown. Source data

**Extended Data Fig. 6:**
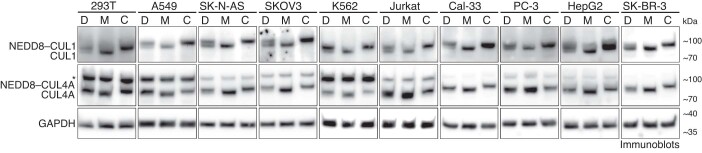
Cellular neddylation levels across cell lines. Overview of cellular neddylation levels for CUL1 and CUL4A after 2 h treatment with DMSO (D), MLN4924 (M), and CSN5i-3 (C) for 293T, A549, SK-N-AS, SKOV3, K562, Jurkat, CAL-33, PC-3, HepG2, and SK-BR-3 as determined by immunoblotting. GAPDH serves as the sample processing control. Slower migrating forms of cullins lost by MLN4924 treatment are interpreted as NEDD8-modified, whereas faster-migrating forms of cullins increased by MLN4924 treatment are interpreted as unneddylated. *—band cross-reacting with anti-CUL4 antibody. Shown results are representative of two independent experiments. Source data

We next addressed the fundamental question of whether active CRL repertoires vary in different cell types by quantitatively comparing cellular landscapes of neddylated CRLs without endogenous tagging of cullins. Using the proteomics pipeline, we probed neddylated CRL repertoires across a panel of ten cell lines, derived from kidney, tongue, brain, blood, bone, lung, ovary and prostate. To normalize for intrinsic differences (Extended Data Fig. 6), we compared the relative loss of SBMs in N8C_Fab3b IPs arising from 2-h MLN4924 treatment. Remarkably, the relative levels of 83 SBMs changed substantially in at least one cell line (Fig. 6a). Several SBMs, for example, BTRC, KLHL12 and CRBN, show large variations in neddylated CRL occupancies in different lines, while VHL was highly associated except in CAL33 cells (Fig. 6b–d).

Can pre-assembly into an active CRL impact response to a degradation signal? We tested this by examining dBET6-induced degradation of BRD4, mediated by neddylated CRL4^CRBN^ (Fig. 6c). We selected this system based on the following criteria: (1) CRL4^CRBN^ is a predominant E3 employed in targeted protein degradation^32,46^; (2) it is regulated by NEDD8 in a multimodal manner^21^; and (3) dBET6 is a designed bivalent degrader molecule harnessing CRBN whose activity depends on neddylation/deneddylation and CRL assembly/disassembly machineries^21,54^. Four cell lines were selected spanning the range of CRBN assembly with neddylated cullins, high in 293 T and Jurkat cells, lower in SK-N-AS and very low in CAL33. CRBN levels in N8C_Fab3b IPs largely correlated with its expression. However, SK-N-AS and CAL33 cells showed nearly identical CRBN quantities but different degrees of assembly into active CRL complexes, indicating that the formation of neddylated CRL4^CRBN^ also depends on factors beyond protein levels (Fig. 6d). Degradation efficiency correlated most strongly with the degree of CRBN assembly into a neddylated CRL as determined by our probe (Fig. 6e).

**Extended Data Fig. 7:**
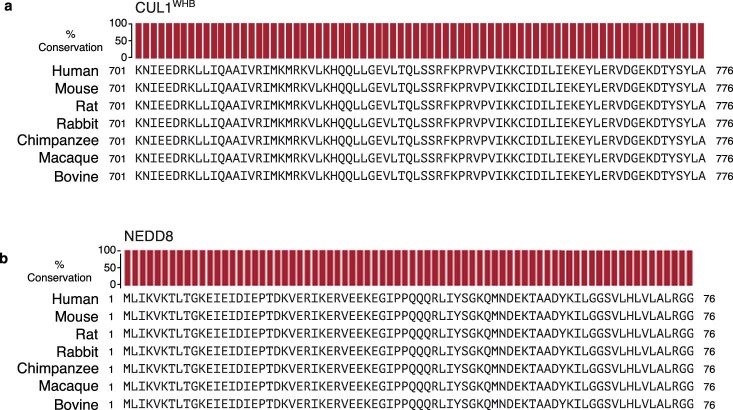
Sequences of the CUL1 WHB domain and NEDD8 are conserved across mammals. **a**, Sequence alignment of the CUL1 WHB domain from human, mouse, rat, rabbit, chimpanzee, macaque, and bovine. Red bars indicate percent sequence conservation. **b**, Sequence alignment for NEDD8 from organisms as in a.

**Extended Data Fig. 8:**
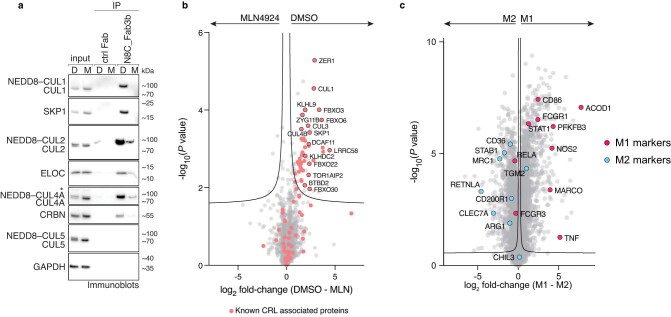
Profiling neddylated CRL repertoires in mouse cells. **a**, Immunoblots are shown after IP using N8C_Fab3b or a ctrl Fab from 3T3 murine cells treated with DMSO (D) or MLN4924 (M) for 2 h. The CRL components and the GAPDH sample processing control detected in immunoblots are indicated. Slower migrating forms of cullins lost upon MLN4924 treatment are interpreted as NEDD8-modified, whereas faster-migrating forms of cullins are interpreted as unneddylated. Shown results are representative of two independent experiments. **b**, Volcano plot of N8C_Fab3b profiling of mouse bone marrow derived macrophages after treatment with DMSO or MLN4924 (MLN) for 2 h (n = 3 biological independent samples). Known CRL and ubiquitin-proteasome system components are indicated in red. Selected proteins of interest are shown are labeled with their gene names and the curve for 5% false discovery rate (FDR) (FDR controlled, two-sided *t*-test, randomizations = 250, s0 = 0.1) is shown. **c**, Volcano plot comparing the total proteome for mouse bone marrow derived macrophages activated to M1 or M2 by treatment with LPS or a combination of IL-4 and IL-13, respectively (based on results shown for Fig. 6g, n = 4 biological independent samples). Marker proteins for the M1 and M2 activation states are highlighted in pink and light blue, respectively. The curve for 5% FDR (FDR controlled, two-sided *t*-test, randomizations = 250, s0 = 0.1) is shown. Source data

Conservation of the cullin WHB domain and NEDD8 sequences suggested that our profiling method could be extended to cells from other mammals (Extended Data Fig. 7a,b). Indeed, N8C_Fab3b IPs also enriched mouse cullins, adapter proteins and SBMs in a neddylation-dependent manner (that is, MLN4924-sensitive; Extended Data Fig. 8a). This afforded the opportunity to probe active CRL repertoires from cells derived from a primary source (Extended Data Fig. 8b). Therefore, we investigated differences across macrophage activation, a robust ex vivo model of cell state changes related to anti-microbial (M1) or anti-helminth and tissue reparative (M2) functions^55,56^. Mouse macrophages were differentiated from bone marrow progenitors with CSF1 and left unstimulated (M0) or stimulated with LPS or IL-4 plus IL-13 to form M1 or M2 activation states, respectively (Fig. 6f and Extended Data Fig. 8c). Total proteome analyses showed that M1 and M2 macrophages have generally similar levels of CRL components. However, profiling with N8C_Fab3b revealed considerable differences in their neddylated CRL repertoires (Fig. 6g). Interestingly, the 37 SBMs found to differ between the M1 and M2 activation states include six of the eight known to bind ‘C-degrons’, which are specific sequences at protein C-termini^57,58^. C-degrons are thought to be generated in stress conditions that trigger mistranslation or proteolytic cleavage. Notably, APPBP2, FEM1C, KLHDC2 and KLHDC3 are associated with the degradation of selenoproteins prematurely truncated in selenium-limiting conditions, while KLHDC10 facilitates nascent chain clearance from stalled ribosomes^58,59^. The redox sensing SBMs KEAP1 (refs. ^60,61^) and FEM1B^62,63^ also stand out as maintained between M0 and M2 but relatively decreased among the CRLs active in M1, consistent with distinct metabolism and roles of the different macrophage states in inflammatory responses^55,56,64^. Our data imply overall that distinct CRLs are activated during stress pathways differentially deployed by M1 or M2 activation states.

## Discussion

In this work, we generated eight affinity reagents selectively binding neddylated cullins in solution and immunoblots, six of which specifically IP neddylated cullins and protect CUL1 and/or CUL2 from CSN-mediated deneddylation (Figs. 1 and 2). We deeply characterized and developed one, N8C_Fab3b, which recovers neddylated CUL1, CUL2, CUL3 and CUL4 and their associated proteins in IPs (Fig. 4d).

N8C_Fab3b is not simply a coincidence detector of NEDD8 and a cullin; it binds the active arrangement (Fig. 3). While this conformation has only been structurally visualized for CUL1-based complexes with UCEs^11,12^, the binding of N8C_Fab3b provides biochemical evidence that this active conformation is conserved for NEDD8-linked CUL1–CUL4. Remarkably, this Fab leaves NEDD8’s I44-patch exposed to bind ARIH1 during ubiquitylation (Fig. 3d and Extended Data Fig. 2f). Unlike E2 enzymes, which disengage after performing ubiquitylation^65^, ARIH1 alone retains high affinity for neddylated CRLs^10^. ARIH1 copurifies with numerous CRLs in a neddylation-dependent manner and mediates their biological regulation^10,27^. Thus, the capacity to accommodate ARIH1 could be important for surveying cellular landscapes of neddylated CRLs, and our mechanistic data showed that N8C_Fab3b could achieve this feat. This also showcases the ability of an affinity reagent to dually recognize a ubiquitin-like protein and its target, which is a hallmark feature of downstream effectors^11,12,66–69^, while simultaneously still permitting such an effector to bind.

Performing IPs with N8C_Fab3b in various settings revealed fundamental features of the neddylated CRL network. For example, there is not a singular effect of inhibiting neddylation or deneddylation on SBM association across the CRL system (Fig. 4d). These data highlight the diversity of mechanisms controlling assembly, disassembly, activation and deactivation of different CRLs. Levels of most SBMs detected in N8C_Fab3b IPs conformed to expectations, decreasing upon MLN4924 treatment. However, for some, association with neddylated cullins is impaired by both MLN4924 and CSN5i-3, whereas others increased with MLN4924 and decreased with CSN5i-3 treatments. The former class likely comprises SBMs subject to autoubiquitylation-dependent degradation, which would be lost upon MLN4924 eliminating neddylation, and CSN5i-3 promoting neddylation-dependent degradation^21^. Meanwhile, the latter class highlights the multifarious regulation of CRL neddylation status, which for some SBMs is additionally influenced by interconversion between alternative assemblies^26,28^.

As previous studies showed that the repertoire of SBMs copurifying with endogenously tagged CUL1 or CUL4 shifts upon cell treatment with various extracellular stimuli^22,24^, we considered that N8C_Fab3b IPs could be used to identify pathways stimulated by signals but without requiring endogenous tagging. Because Fab-binding interferes with CSN-mediated deneddylation and substrate ubiquitylation by UBE2D-family E2s, its expression in cells could impact cellular regulation in complicated ways. Nonetheless, N8C_Fab3b proved useful for affinity purification from lysates. Although the steric bulk of the Fab might lead to some CRL complexes being preferentially recognized over others, our data show that N8C_Fab3b IPs can identify pathways stimulated by diverse signals without requiring endogenous tagging. This extends concepts from studies focusing on complexes with a single endogenously tagged cullin^22,24^; we show the vast network of CRL1, CRL2, CRL3 and CRL4 complexes responds to small molecule degraders and signaling pathways by precise rearrangement.

Our robust and portable proteomics pipeline employing N8C_Fab3b can identify SBMs—and in some cases their substrates—responding to signals. Moreover, for the TNF stimulus, the pipeline also identified the kinase producing a substrate phospho-degron required for SBM binding, and components of the transcriptional complex regulated by the neddylated CRL (Fig. 5). Thus, our workflow can illuminate entire signaling pathways. Although neddylated CRL1^SKP2^ was known to assemble with the cyclin–CDK2–CKSHS1 kinase phosphorylating its substrate p27 (refs. ^6,12^), our results suggest kinase–substrate–E3 ligase signaling complexes may be more common than previously appreciated.

A key feature of our pipeline is that it can be generically applied to mammalian systems. We found striking variation in the repertoires of more than 70 SBMs across different cell lines (Fig. 6a). Pursuing targeted protein degradation mediated by one SBM (CRBN) showed an overall correspondence between its protein levels and degradation efficiency. However, targeted protein degradation efficiency correlated better with CRBN association with neddylated cullins. Furthermore, we could apply the probe to gain new insights into a system not readily amenable to endogenous tagging, originating from a mouse. We discovered that neddylated CRL repertoires vary across macrophage activation states, with noteworthy differences in E3s associated with quality control and selenium and redox stress responses, especially those recognizing C-degrons (Fig. 6g). Thus, not only do CRL networks dynamically rearrange to drive cellular signaling, but we propose that CRL repertoires may also adjust to resolve stresses arising from toxic effectors such as those required for macrophage activities that include microbial killing, efferocytosis and tissue repair^55,56,64^.

Finally, this study highlights the potential for using binders recognizing a specific biologically-relevant conformation, or an activating PTM and its target, to select active complexes from among broader pools of constituent molecules. We show the utility of conformation-specific affinity probes to ensnare E3 complexes lacking residues easily targeted by reactive chemical moieties. Our approach selectively targeting a site-specific modification and conformation enables new insights into dynamic E3 ligase systems and targeted protein degradation pathways.

## Methods

### Cloning, protein expression and purification

All proteins are of human origin.

#### Cullin expression and purification

Soluble versions of cullin C-terminal regions bound to RBX1 were used as baits for Fab selection^7,10^. For CUL1, the C-terminal region corresponds to residues 411–776 (with solubilizing substitutions L421E, V451E, V452K and Y455K); for CUL2, residues 380–745 (with solubilizing substitutions L390E, T420E, V421K and Y424K); for CUL3, residues 382–768 (with solubilizing substitutions V417K and L418K); for CUL4A, residues 400–759 (with solubilizing substitutions L408K, I438D, L439D and F442Y); and for CUL5, residues 411–780 (with solubilizing substitutions L407E, L439K and V440K). For crystallization, a Thrombin cleavage site was inserted between K676 and N677 of CUL1 C-terminal region. All cullin C-terminal regions were N-terminal GST-fusions co-expressed with MBP-TEV-RBX1 in *Escherichia coli* BL21Gold (DE3) cells. Cells were grown to an OD_600_ of 0.8 and induced with 0.6 μM isopropyl-β-d-thiogalactopyranoside (IPTG) for 17 h at 16 °C. Proteins were purified by glutathione affinity with wash buffer (50 mM Tris pH 8, 200 mM NaCl, 5 mM DTT) and elution buffer (50 mM Tris pH 8, 200 mM NaCl, 5 mM DTT and 10 mM reduced glutathione). Pooled fractions were cleaved with 1:100 TEV overnight at 4 °C. CUL C-terminal region–RBX1 complexes were purified by cation exchange with bump elution buffer (50 mM HEPES pH 7, 1 M NaCl and 1 mM DTT).

For expression of full-length cullin–RBX complexes, wild-type CUL1, CUL2, CUL3, CUL4A, CUL5, GST–TEV–RBX1 (residues 5-C) and GST–TEV–RBX2 (residues 5-C) were cloned into pFastBac. CUL1, CUL2, CUL3 and CUL4A were co-expressed with the GST-tagged RBX1 partner in High-Five (Hi5) insect cells by coinfection with separate baculoviruses produced in SF9 cells. CUL5 was co-expressed with GST-tagged RBX2. Cullin–RBX complexes were batch purified by glutathione affinity chromatography, cleaved with TEV protease (unless indicated otherwise) and further purified by ion exchange and SEC.

#### Fab expression and purification

Heavy and light chains contained an N-terminal periplasmic leader sequence (from *E. coli* heat-stable enterotoxin II) and C-terminal peptide tags, a FLAG tag on the light chain and a hexahistidine tag on the heavy chain. Fabs were expressed in *E. coli* Rosetta (DE3) cells by bicistronic expression using a pET vector. Cells were grown in Terrific Broth to an OD600 of 0.8 and induced with 1 mM IPTG for 17 h at 18 °C. Pellets were resuspended in HBS (50 mM HEPES pH 7.4 and 300 mM NaCl), with 10 mM imidazole, lysed by sonication, subjected to Ni^2+^-AP and eluted with HBS, 250 mM imidazole. Fabs were further purified by ion exchange and selected fractions of buffer were exchanged into HBS (30 mM HEPES pH 7.4 and 150 mM NaCl) by spin concentration.

For the production of biotinylated Fabs, the hexahistidine tag on the heavy chain was exchanged with an AviTag-hexa-histidine tag with a GS-linker between the heavy chain and the tag. Fabs were then site specifically biotinylated at the AviTag in vitro using BirA ligase after the initial Ni^2+^-AP step. Fabs were diluted to ~80 µM by the addition of bicine (pH 8.3) to 50 mM, ATP to 10 mM and Mg(OAc)_2_ to 10 mN. Recombinant BirA ligase was added at a molar ratio of 1:100 and the reaction was incubated overnight at 4 °C. Successful biotinylation was tested by binding the Fab to streptavidin and biotinylated Fabs were further purified as described above.

Labeling of N8C_Fab3b with Alexa Fluor 647 was done using Alexa Fluor 647 NHS Ester (Succinimidyl Ester; Thermo Fisher Scientific, A20006) following the manufacturer’s instructions.

#### Other proteins

Ubiquitin, NEDD8, NAE1 and UBE2F were expressed in BL21 Gold (DE3) *E. coli* as GST–Thrombin and UBE2D3, UBE2L3 and ARIH1 as GST–TEV^11,12,35^. His–MBP–TEV–BTRC^(175-C)^ and GST–TEV–FBXW7^(263-C)^ were co-expressed with SKP1 in BL21 Gold (DE3) *E. coli*^11^. GST–3C–IKZF1^ZF2^–Strep (residues 141–169, K157R, K165R, 140K) was produced in BL21 Gold (DE3)^11^. UBE2M-His and GST–TEV–UBA1 were expressed in Hi5 cells^11^. CSN was prepared by co-expression of all subunits^18^. His–TEV–DDB1 and GST–TEV–CRBN were co-expressed in Hi5 cells^11^. Proteins were either batch purified using glutathione or Ni-NTA affinity resin, followed by proteolytic cleavage with the indicated protease and subsequently further purified by ion exchange and SEC^12,35^.

For neddylation of cullins (CUL1–CUL4), 16 µM cullin was modified in 30 mM Tris pH 7.6, 50 mM NaCl, 10 mM ATP and 10 mM MgCl_2_ using 80 µM NEDD8, 4 µM UBE2M and 700 nM NAE1 for 8 min at room temperature and the reaction was quenched with 10 mM DTT. For neddylation of CUL5, UBE2F was used instead of UBE2M. For fluorescent labeling of ubiquitin (*Ub), the N-terminal RRASV sequence was replaced with RRACV and labeling was performed using fluorescein-5-maleimide (Thermo Fisher Scientific, 62245) following manufacturer’s instructions. For fluorescent labeling of NEDD8 (^Cy5^NEDD8), a Cy5-labeled peptide (Cy5-(PEG)_5_-LPETGG) was conjugated to NEDD8 in a sortase-mediated reaction using 50 µM NEDD8, 10 µM Sortase and 200 µM peptide in 50 mM Tris pH 7.5, 150 mM NaCl and 10 mM CaCl_2_.

### Selection of Fabs by phage display

Phage selections were performed using established protocols^70^. Purified NEDD8–CUL^CTD^–RBX1 complexes (5 µg ml^−1^) were coated on 96-well MaxiSorp plates (Thermo Fisher Scientific, 12565135) overnight at 4 °C. A phage-displayed Fab library was cycled through five rounds of binding selections with the immobilized proteins to enrich for antibodies for neddylated cullins. To eliminate phage that bound nonspecifically, phage was preincubated sequentially on plates coated with neddylated and unneddylated cullins (rounds 1–5). After five rounds of selection, specific binding clones were detected by clonal phage ELISA and identified by DNA sequencing.

### Affinity maturation

Affinity-matured libraries were constructed using oligonucleotide-directed mutagenesis (Kunkel mutagenesis method)^71^. CDR-L3 and CDR-H3 of the phagemid template were mutated using degenerate oligonucleotides containing ratios of 70% of the WT nucleotide and 10% of each of the other three nucleotides (that is, soft randomization strategy). The diversity of the library was 1.5 × 10^9^, with an incorporated diversity of 64% and 74% in CDR-L3 and CDR-H3, respectively.

### ELISAs

Phage and protein ELISAs were carried out on immobilized proteins. Proteins (2 µg ml^−1^) were coated on 384-well MaxiSorp plates (Thermo Fisher Scientific, 12665347) overnight at 4 °C. Phage and protein binding was detected with anti-M13-HRP antibody (1:5,000; GE Healthcare, 27-9421-01) and anti-Kappa-HRP (1:5,000; Southern Biotech, 2060-05), respectively. The binding affinities of the purified Fab proteins were determined as EC_50_ values, defined as the concentration of Fab concentration at which 50% of binding was observed by ELISA. EC_50_ values were calculated with the GraphPad Prism software using a nonlinear regression model algorithm.

### Cell culture

All cells were cultured with 10% fetal bovine serum (Gibco, A3160802), 4 mM GlutaMAX (Gibco, 35050038), 1 mM sodium pyruvate (Gibco, 11360070; 100 units per ml penicillin) and 100 µg ml^−1^ streptomycin (Gibco, 15140122) at 37 °C and 5% carbon dioxide. For CAL33, 293T, SK-N-AS, A549 and Hep G2 cells, Dulbecco’s Modified Eagle Medium (DMEM; Gibco, 11960044) was used. K562s were cultured in Iscove’s Modified Dulbecco’s Medium (Gibco, 12440053), Jurkat cells in RPMI 1640 (Gibco, 72400021), SK-OV-3 cells in McCoy’s 5A (modified) Medium (Gibco, 26600023) and PC3 cells in Ham’s F-12 Nutrient Mixture (Gibco, 21127022). All cells were periodically tested for mycoplasma using MycoAlert (Lonza, LT07-318) kits.

### Immunoblots

Immunoblots were performed using peroxidase-conjugated secondary antibodies together with SuperSignal West Pico PLUS Chemiluminescent Substrate (Thermo Fisher Scientific, 34580). Blots were imaged on an Amersham ImageQuant 800 (Cytiva). Secondary antibodies used for detection were goat anti-rabbit-HRP (1:5,000; Thermo Fisher Scientific, 31460), donkey anti-mouse-HRP (1:5,000; Jackson ImmunoResearch, 715-035-150) and Streptavidin-HRP (1:5,000; Cell Signaling Technology, 3999). Primary antibodies used in this study are anti-CUL1 (1:1,000; Santa Cruz Biotechnology, sc–17775), anti-CUL2 (1:1,000; Abcam, ab166917), anti-CUL3 (1:1,000; Bethyl Laboratories, A301-109A), anti-CUL4A (1:1,000; Bethyl Laboratories, A300-739A), anti-CUL5 (1:1,000; Abcam, ab184177), anti-SKP1 (1:1,000; Cell Signaling Technology, 2156), anti-BTRC (1:1,000; Cell Signaling Technology, 4394), anti-ELOC (1:1,000; Biolegend, 613101), anti-DDB1 (1:1,000; Abcam, ab109027), anti-CRBN (1:1,000; Sigma, HPA045910), anti-BRD4 (1:1,000; Cell Signaling Technology, 13440) and anti-GAPDH (1:5,000; Cell Signaling Technology, 2118). For the detection of recombinant cullins with the N8C_Fabs, 200 ng of either neddylated or unneddylated CUL1-5 were probed for using 2 µg ml^−1^ biotinylated Fabs as the primary binder and Strepavidin-HRP as a secondary binder. For all immunoblots, PVDF membranes were used, and blocking and incubation with the secondary antibodies (except Strepavidin-HRP) were performed in 5% nonfat dry milk in TBST (20 mM Tris pH 7.4, 150 mM NaCl, 0.1% Tween-20), while incubation with primary antibodies and Strepavidin-HRP was performed in 5% bovine serum albumin in TBST. The brightness and contrast of raw images were adjusted in Fiji.

### IP experiments

To preserve the active CRL repertoire of cells for IP, cells were exposed to an ‘N8-block’ treatment (3 min treatment with 1 µM MLN4924 (MedChemExpress, HY-70062) and 1 µM CSN5i-3 (MedChemExpress, HY-112134) in media)^24^ during the process of collection, after which they were washed in PBS (Gibco, 14190094) and lysed in lysis buffer (25 mM HEPES pH 7.4, 100 mM NaCl, 0.5% IGEPAL CA-63, 5% glycerol, 1× protease inhibitor (Sigma-Aldrich, 11836145001), 1 µM MLN4924 and 1 µM CSN5i-3). Lysates were homogenized by brief sonication (10 s, 1 s on/off, 10% amplitude, Bandelin Sonopuls HD 4200, TS 103) and cleared by centrifugation at ~20,000*g* for 3 min at 4 °C. High Capacity Magne Streptavidin Beads (Promega, V7820) were coated with indicated biotinylated Fabs following manufacturer’s instructions. The equivalent of 6 µl of bead slurry of the Fab-coated beads was added to the cleared cell lysates and incubated for 45 min at 4 °C while rotating. Beads were washed twice with lysis buffer, twice with wash buffer (lysis buffer without IGEPAL CA-63) and twice with HBS (25 mM HEPES pH 7.4 and 150 mM NaCl). After the last wash, all buffer was removed and beads were resuspended in reducing sample buffer and analyzed by immunoblotting.

### Flow cytometry

K562 cells were plated in 96-well plates and treated with indicated concentrations of MLN4924 for 2 h. Cells were washed two times with PBS and then fixed with paraformaldehyde (Thermo Fisher Scientific, 28908) for 10 min at room temperature, followed by cell permeabilization using ice-cold methanol at −20 °C for 1 h and two washes with PBS–BSA (PBS containing 0.5% BSA and 0.1% sodium azide). Cells were incubated with ~0.002 mg ml^−1^ N8C_Fab3b-Alexa Fluor 647 in PBS–BSA for 1 h at room temperature while shaking. Samples were washed twice with PBS–BSA and measured on an Attune NxT (Thermo Fisher Scientific) flow cytometer with 10,000 total events being collected (Supplementary Fig. 1). Mean fluorescence intensities (MFIs) were extracted using FlowJo (BD Biosciences) and values normalized to the minimal and maximal MFI as averaged across replicates. Samples were plotted in Prism 9 (GraphPad) and the dose–response curve was generated using the ‘sigmoidal dose–response’ analysis function.

### Biochemical assays

#### CSN deneddylation assays

Here 100 nM CUL1 or CUL2 neddylated with Cy5-NEDD8 was incubated for 10 min on ice with 10× molar excess of Fab in reaction buffer containing 50 mM Tris pH 7.5, 50 mM NaCl and 2.5 mM MgCl_2_. The reactions were initiated by the addition of 10 nM CSN and proceeded at room temperature until quenched at indicated time points in SDS sample loading buffer. Reaction products were separated by SDS–PAGE and the fluorescence signaling was detected using an Amersham Typhoon imager (Cytiva).

#### Substrate ubiquitylation assays

Experiments were performed in a pulse-chase format to avoid the effects of the UBA1-dependent formation of the E2~Ub intermediate. For CRL1^BTRC^-dependent ubiquitin transfer from UBE2D3 to NFKBIA, the pulse reaction to produce the thioester-linked UBE2D3~Ub intermediate contained 10 µM UBE2D3, 15 µM fluorescent ubiquitin and 0.2 µM UBA1 in reaction buffer containing 50 mM Tris pH 7.5, 50 mM NaCl, 2.5 mM MgCl2 and 1.5 mM ATP and was incubated for 10 min at room temperature. The reaction was quenched by the addition of 25 mM EDTA and diluted to a final concentration of 100 nM UBE2D3 in 25 mM MES pH 6.5 and 150 mM NaCl. The chase reaction mix consisted of 400 nM CRL1 (NEDD8–CUL1–RBX1–SKP1–BTRC) and 1 µM substrate (phosphorylated peptide derived from NFKBIA, KKERLLDDRHD(pS)GLD(pS)MKDEE)^11^ in 25 mM MES pH 6.5, 150 mM NaCl incubated on ice. To test the effects of Fab binding on CRL reactivity, Fab was added to the chase mix at indicated molar excess as compared to CRL. The quenched pulse reaction mix was added to the chase reaction mix at a 1:1 ratio on ice, resulting in final concentrations of 50 nM UBE2D3~Ub and 200 nM neddylated CRL1^BTRC^. Samples were taken at indicated time points and the reaction was stopped by the addition of 3× SDS–PAGE samples buffer. Reaction products were separated by SDS–PAGE and the fluorescence signaling was detected using an Amersham Typhoon imager (Cytiva).

For ARIH1/CRL1^FBXW7^-dependent ubiquitin transfer from UBE2L3 to CCNE, reaction conditions were similar to the same concentrations for CRL and pulse mix being used. In addition to the neddylated CRL, 400 nM ARIH1 was added to the chase reaction mix (200 nM final concentration, equimolar with the CRL) and 2 µM phosphorylated peptide derived from CCNE (KAMLSEQNRASPLPSGLL(pT)PPQ(pS)GRRASY)^10^ was used as the substrate.

Reactions with CRL4^CRBN^ were performed similarly with final concentrations of 100 nM E2~Ub, 500 nM CRL4^CRBN^ (and ARIH1 when indicated), 5 µM pomalidomide and 2.5 µM IKZF ZF2 as a substrate. Reactions were performed at room temperature and samples were taken at indicated time points.

#### NFKBIA substrate ubiquitylation assay in presence of CSN

Reactions were performed as described above for ARIH1/CRL1^FBXW7^ with minor modifications. Chase mix was incubated for 10 min on ice with indicated molar excess of N8C_Fab3b as compared to CRL1 before the addition of 200 nM CSN (equimolar with CRL1) where indicated followed by five more minutes of incubation on ice. Pulse mix was then added and samples were taken at indicated time points.

### Bio-Layer Interferometry measurements

Bio-Layer Interferometry measurements were performed on an Octet K2 system (ForteBio) at 30 °C with shaking at 1,000 rpm. Concentrated proteins were diluted into BLI reaction buffer (25 mM HEPES pH 7.5, 150 mM NaCl, 0.1 mg ml^−1^ BSA and 0.01% Tween-20). For all measurements, anti-GST biosensors (Sartorius, 18-5097) were used. GST RBX1–CUL1–NEDD8 was served as the ligand immobilized on the biosensors and His-tagged N8C_Fab3b was served as the analyte. For the measurement, six dilutions of N8C_Fab3b ranging from 200 to 6.25 nM were applied. Raw data were processed by the Octet Data Analysis HT software (Release 11.1). Both association and dissociation were analyzed assuming a 1:1 binding model, performing a global (group) fitting with linked *R*_max_ values. The dissociation constant (*K*_*D*_), association rate (*k*_*a*_) and dissociation rate (*k*_dis_) were reported as calculated by the software. Processed data and fitted curves were plotted in Prism (v9).

### Complex formation and purification for crystallization

Here 16 µM CUL1^CTD^ (Thrombin 676/677)–RBX1 was neddylated as described above, and the reaction was quenched with 10 mM DTT. Also, 500 µg ml^−1^ of Thrombin and 2.5 mM CaCl_2_ were added for 1 h at 16 °C, and N8–CUL1^WHB^ was purified away from the remaining CUL1^CTD^ and RBX1 by two rounds of SEC using a Superdex 200 into a final buffer of 25 mM Tris pH 7.6 and 200 mM NaCl. Equimolar concentrations of the NEDD8–CUL1^WHB^ and N8C_Fab3b were incubated on ice for 15 min, and the complex was purified by SEC) using a Superdex 200 into a final buffer of 25 mM Tris pH 7.6 and 200 mM NaCl.

### Crystallization

N8C_Fab3b–NEDD8–CUL1^WHB^ at 12.5 mg ml^−1^ was mixed in a ratio of 1:1 with good buffer (2.1 M AmSO_4_, 100 mM citrate pH 6, 10 mM tris(2-carboxyethyl)phosphine (TCEP)) and crystals of N8C_Fab3b-bound NEDD8–CUL1^WHB^ were obtained by hanging drop vapor diffusion at room temperature.

### Crystallographic data collection and structure determination

A crystallographic dataset was collected at the NE-CAT beamline (24-ID-E) of the Advanced Photon Source (APS). Datasets were integrated and scaled using XDS (version: 3 November 2014)^72^. Crystals formed in the P2_1_2_1_2_1_ spacegroup with unit cell edges *a* = 101.9 Å, *b* = 107.2 Å and *c* = 185.1 Å with two molecules of Fab-bound NEDD8–CUL1^WHB^ per asymmetric unit. The structure of N8C_Fab3b-bound NEDD8–CUL1^WHB^ was solved by molecular replacement with Phaser (v.2.5.6)^73^ using the structure of HER2 bound to Herceptin (Protein Data Bank (PDB): 1N8Z). The coordinates of the isolated Fab were used as the search model. Multiple rounds of rebuilding and crystallographic refinement were performed using COOT (v.0.8)^74^ and Phenix (v.1.9-1692)^75^. Diffraction and refinement statistics as well as structural quality measurements are listed in Supplementary Table 1. Analysis and visualization of structures were performed in UCSF ChimeraX (v.1.2.5)^76^.

### AP–MS

For AP–MS, experiments around 75 µl of compacted cells were lysed in 400 µl lysis buffer. IP was performed similarly as described above, including an additional lysate clearing step by filtration using 0.22 µm spin filters (Corning, 8161). After performing IPs, the beads were resuspended in 45 µl denaturing lysis buffer (100 mM Tris pH 8.5 and 1% SDC) and boiled for 5 min at 98 °C. 2-Chloroacetamide and TCEP were added to final concentrations of 40 mM and 10 mM, respectively, and samples were incubated at 45 °C for 5 min. Trypsin (Sigma-Aldrich, T6567) and LysC (FUJIFILM Wako, 125-05061) were added at 1:100 w/w and samples were digested overnight at 37 °C with agitation (1,200 rpm). Digested samples were cleaned up using SDB-RPS StageTips^77^. Samples were diluted 5× with loading buffer (1% trifluoroacetic acid (TFA) in isopropanol) and loaded onto the StageTips. Tips were then washed once with loading buffer and twice with 200 µl StageTips wash buffer (0.2% TFA/2% acetonitrile (ACN)). Samples were eluted with 60 µl of 1.25% ammonium hydroxide in 80% ACN and dried using a SpeedVac centrifuge. Dried samples were recovered in buffer A* (0.2% TFA and 2% ACN) and normalized to a peptide concentration of 0.1 mg ml^−1^-based absorbance at 280 nm.

### Total proteome analysis

For total proteome analysis, cells were washed four times in PBS before the addition of denaturing lysis buffer. Lysates were incubated at 98 °C for 5 min, sonicated (3 s, 20% amplitude) and cleared by centrifugation. Sample digestion and clean-up were performed as described above.

### LC–MS/MS measurements

Peptides were loaded onto a reverse-phase column (50 cm length, 75 μm inner diameter, packed in-house with ReproSil-Pur C18-AQ 1.9 μm resin (Dr. Maisch HPLC GmbH)). The column was maintained at a temperature of 50 °C using a homemade column oven. Nano-flow liquid chromatography was performed using an EASY-nLC 1200 system directly coupled to the mass spectrometer (Orbitrap Exploris 480, Thermo Fisher Scientific) via a nano-electrospray source. Per measurement, 200 ng of peptides were loaded and separated using a binary buffer system consisting of buffer A (0.1% formic acid (FA)) and buffer B (0.1% FA, 80% ACN). IP samples were separated at a flow rate of 300 nl/min using a 60-min gradient starting at 5% buffer B, followed by a stepwise increase to 30% in 45 min, 65% in 8 min and 95% in 2 min, staying at 95% for 5 min. Total proteome samples were separated using a 120-min gradient starting at 5% buffer B, followed by a stepwise increase to 30% in 90 min, 65% in 16 min and 95% in 4 min, staying at 95% for 10 min. MS data were collected in DIA mode consisting of one MS1 full scan followed by 32 MS2 windows (Supplementary Data). MS1 full scans (300–1,650 m/z range) were acquired at a resolution of 120,000 at 200 m/z with the automatic gain control (AGC) target set to 3 × 10^6^ at a maximum injection time of 20 ms (60 min gradient) or 60 ms (120 min gradient). Each MS2 scan was collected at a resolution of 30,000 at m/z 200 with AGC adjusted to 10 × 10^6^, maximum injection time set to 54 ms and the normalized HCD collision energy at 28% (60 min gradient) or 27% (120 min gradient). The default charge state was 2 and RF lens was set to 40%. All spectra were recorded in profile mode.

### MS data analysis

DIA raw files were processed using Spectronaut (version 15, Figs. 4b and 6a,b and Extended Data Fig. 5a; version 16, Figs. 4c,d, 5b,d,f,h and 6d–g, Extended Data Fig. 5b–f and Extended Data Fig. 8b,c)^78^ using default settings for directDIA. The Spectronaut results are provided in Supplementary Data. Data were analyzed using the Perseus software package (v.1.6.7.0). Protein intensities were log_2_-transformed, and the datasets were filtered to contain a minimum of 50% valid values in at least one experimental condition. Missing values were imputed using a normal distribution with a width of 0.3 and a downshift of 1.8. Intensities for CUL1–CUL4, their adapter proteins and known SBMs, as well as other proteins known to associate with CRLs (Supplementary Data) were extracted and when indicated filtered for an average coefficient of variation within experimental conditions of 15%. When singular values of the log_2_ fold change are shown, it was calculated by subtracting the average of the log_2_-transformed protein group intensities of the replicates of one experimental condition from the other. For Fig. 6b, log_2_(fold changes) were calculated by subtracting the log_2-_transformed protein group intensities of individual replicates between experimental conditions. Replicates of DMSO- and MLN4924-treated cells were paired based on the order they were measured in. For generating Volcano plots, the function included in Perseus was used, which was also used to produce the curves highlighting a 5% false discovery rate (FDR; s0 = 0.1). For the cell line panel, raw files were processed together and SBMs showed significant enrichment (*t* test, FDR = 5%, s0 = 0.1) between the DMSO- and MLN4924-treated samples in at least one cell line extracted.

### CRL repertoire

For Fig. 4b, IP–MS experiments were performed as described above with indicated Fabs as compared to a control Fab bearing wild-type CDRs (Extended Data Fig. 1b). For Fig. 4c,d, 293T cells were treated for 2 h with a DMSO control, 1 µM MLN4924 or 1 µM CSN5i-3, and IP–MS experiments were performed as described above. For Fig. 4c, principal component analysis was performed using the Perseus function with the log_2_-transformed protein group intensities of all in the experiment-identified protein groups serving as the input. Each dot represents an individual biological replicate.

### Profiling CRL repertoire changes

With CRL substrate stability strongly depending on the presence of substrate, we thought to prevent substrate degradation and complex dissociation within cells. To that extent, we treated cells with 10 µM of the proteasome inhibitor MG132 (MedChemExpress, HY-13259) and 10 µM of the VCP inhibitor CB-5083 (MedChemExpress, HY-12861) for 5 min before treatment of cells with the stimulus of interest. To validate our workflow, we used treatment with the molecular glue Indisulam that was previously reported to reshape the CRL4 network^24^. 293T cells were treated with 2 µM Indisulam (MedChemExpress, HY-13650) for 1 h and then collected and prepared for AP–MS experiments as described above. To see whether this reshaping of the CRL network extends to other degraders and CRLs using different cullin backbones, we next tested the bivalent degrader MZ1 hijacking a CRL2 complex by treating 293T cells with 1 µM MZ1 (MedChemExpress, HY-107425) for 1 h. Beyond degraders, many cellular pathways rely on CRL activity. One example is iron homeostasis^52^. We supplied cells with an excess of iron by treatment with 100 µM ferric ammonium citrate (Sigma-Aldrich, F5879) for 90 min to test whether metabolic pathways also reshape the cellular CRL network. Another stimulus known to depend on CRL activity is cytokine signaling. To explore this further, we subjected K562 cells to our AP–MS workflow after serum starving them for 3 h and then treating them with 25 ng ml^−1^ human TNF (PeproTech, 300-01A) for 5 min. *P* values for direct comparisons for SBMs were calculated in GraphPad Prism (v.9) using the *t*-test function (unpaired, two-tailed, 95% confidence level).

### CRBN degradation efficiencies

Indicated cell lines were plated in six-well plates and treated with 0.1 µM dBET6 (MedChemExpress, HY-112588) for indicated time points after which they were lysed in reducing sample buffer. The levels of BRD4 were determined by immunoblotting using GAPDH as a loading control.

### Bone marrow-derived macrophages

Male wild-type C57BL/6N mice were maintained at the animal facility of the Max Planck Institute of Biochemistry under pathogen-free conditions and the use of mice for organ isolation was approved by the Government of Upper Bavaria. Mice were housed in open cages at 22 °C and 55% humidity with a 14-h light cycle/10-h dark cycle. Mouse bone marrow was collected from 8- to 10-week-old mice by flushing femurs and tibiae with PBS. Differentiation into BMDMs was performed by culturing in DMEM with 50 ng ml^−1^ human recombinant CSF1 (produced in-house) for 7 d^79^. For activation, BMDMs were seeded overnight in media containing 100 ng ml^−1^ CSF1 and then stimulated with 10 ng ml^−1^ LPS from *E. coli* O55:B5 (Sigma-Aldrich, L2880) or a combination of 10 ng ml^−1^ mouse recombinant IL-4 (produced in-house) and 10 ng ml^−1^ IL-13 (PeproTech, 210-13) for 24 h. For Extended Data Fig. 8b, BMDMs were treated for 2 h with 1 µM MLN4924 before being processed for active CRL repertoire analysis as described above. BMDMs in their nonactivated and activated states were collected and processed for total proteome (Fig. 6g, left, and Extended Data Fig. 8b) or active CRL repertoire analysis (Fig. 6g, right) as described above.

### Reporting summary

Further information on research design is available in the Nature Portfolio Reporting Summary linked to this article.

## Online content

Any methods, additional references, Nature Portfolio reporting summaries, source data, extended data, supplementary information, acknowledgments, peer review information; details of author contributions and competing interests; and statements of data and code availability are available at 10.1038/s41589-023-01392-5.

### Supplementary information

Supplementary Information Supplementary Table 1 and Supplementary Fig. 1.

Reporting Summary

Supplementary Data Mass spectrometry data tables.

### Source data

Source Data Fig. 1 Unprocessed western blots. Statistical source data.

Source Data Fig. 2 Unprocessed gels.

Source Data Fig. 4 Unprocessed western blots. Statistical source data.

Source Data Fig. 5 Statistical source data.

Source Data Fig. 6 Unprocessed western blots. Statistical source data.

Source Data Extended Data Fig. 2 Unprocessed gels. Statistical source data.

Source Data Extended Data Fig. 3 Statistical source data.

Source Data Extended Data Fig. 5 Statistical source data.

Source Data Extended Data Fig. 6 Unprocessed western blots.

Source Data Extended Data Fig. 8 Unprocessed western blots. Statistical source data.

## Data Availability

Crystallography data were deposited in the RCSB (8CAF.PDB), and proteomics data in the ProteomeXchange Consortium PRIDE database (PXD039649). Plasmids for Fab production are available from Addgene. Source data are provided with this paper.
