# Supplementary material for: Activity-based profiling of cullin–RING E3 networks by conformation-specific probes
Source: Nat Chem Biol. 2023 Aug 31;19(12):1513–23. doi: 10.1038/s41589-023-01392-5 (PMC10667097; doi:10.1038/s41589-023-01392-5)
Supplement: Supplementary file 1 — Supplementary Table 1 and Supplementary Fig. 1. [file 41589_2023_1392_MOESM1_ESM.pdf]

nature chemical biology

Article

https://doi.org/10.1038/s41589-023-01392-5

Activity-based profiling of cullin–RING E3 networks by conformation-specific probes

In the format provided by the authors and unedited

Supplementary Information

Supplementary Table 1: Crystallographic data collection and refinement statistics

Supplementary Figure 1: Example gating strategy for flow cytometry experiments

Supplementary Data 1: Mass spectrometry data tables

Supplementary Table 1: Crystallographic data collection and refinement statistics

	N8C_Fab3b-NEDD8-CUL1^{WHB}
**Data collection**	
Space group	P 21 21 21
Cell dimensions	
a, *b*, *c* (Å)	102.37, 106.87,180.65
α, β, γ (°)	90.00, 90.00, 90.00
Resolution (Å)	90.33 - 2.66 (2.70 - 2.66) *
R_{sym} or R_{merge}	0.06
$I / \sigma I$	1.72 (at 2.65Å)
Completeness (%)	98.3
Redundancy	6.7
**Refinement**	
Resolution (Å)	2.66
No. reflections	56649
R_{work} / R_{free}	0.217/ 0.263
No. atoms	8808
Protein	8808
Ligand/ion	0
Water	0
B-factors	
Protein	77.01
Ligand/ion	
Water	
R.m.s. deviations	
Bond lengths (Å)	0.0094
Bond angles (°)	1.1774

*Values in parentheses are for highest-resolution shell.

Supplementary Figure 1: Example gating strategy for flow cytometry experiments

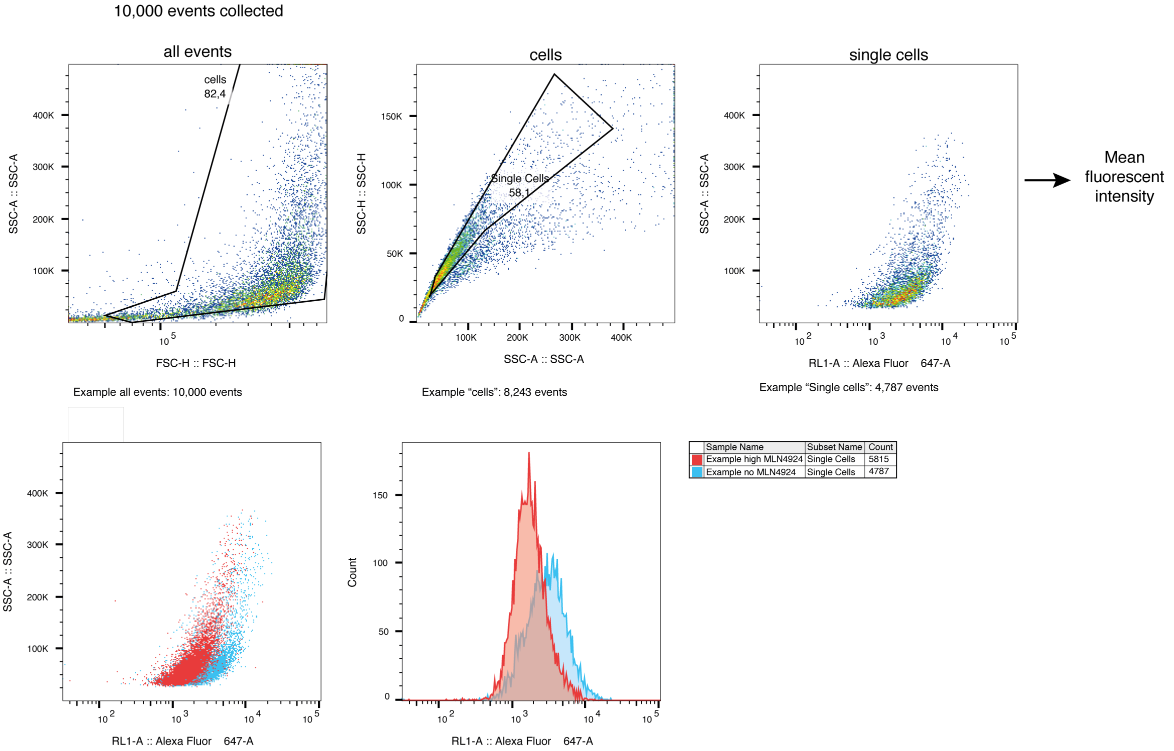

Supplementary Figure 1: Example gating strategy for flow cytometry experiments
Single cells were selected based on FSC-H/SSC-A and subsequent SSC-A/SSC-H plots. A 637 nM laser with a 670/14 gate was used to measure AF647 fluorescence. Mean fluorescent intensity of the "single cells" population was used for generating the plot for Fig. 1f. Bottom plots show comparison of AF647 signal of cells treated with no MLN4924 and 6 μM MLN4924.
