# Supplementary material for: Activity-based profiling of cullin–RING E3 networks by conformation-specific probes
Source: Nat Chem Biol. 2023 Aug 31;19(12):1513–23. doi: 10.1038/s41589-023-01392-5 (PMC10667097; doi:10.1038/s41589-023-01392-5)
Supplement: Supplementary file 2 — Reporting Summary [file 41589_2023_1392_MOESM2_ESM.pdf]

nature portfolio

Corresponding author(s): Sachdev S. Sidhu
Brenda A. Schulman

Last updated by author(s): May 28, 2023

Reporting Summary

Nature Portfolio wishes to improve the reproducibility of the work that we publish. This form provides structure for consistency and transparency in reporting. For further information on Nature Portfolio policies, see our Editorial Policies and the Editorial Policy Checklist.

Statistics

For all statistical analyses, confirm that the following items are present in the figure legend, table legend, main text, or Methods section.

n/a	Confirmed	
☐	☒	The exact sample size (*n*) for each experimental group/condition, given as a discrete number and unit of measurement
☐	☒	A statement on whether measurements were taken from distinct samples or whether the same sample was measured repeatedly
☐	☒	The statistical test(s) used AND whether they are one- or two-sided<br>*Only common tests should be described solely by name; describe more complex techniques in the Methods section.*
☒	☐	A description of all covariates tested
☐	☒	A description of any assumptions or corrections, such as tests of normality and adjustment for multiple comparisons
☐	☒	A full description of the statistical parameters including central tendency (e.g. means) or other basic estimates (e.g. regression coefficient) AND variation (e.g. standard deviation) or associated estimates of uncertainty (e.g. confidence intervals)
☐	☒	For null hypothesis testing, the test statistic (e.g. *F*, *t*, *r*) with confidence intervals, effect sizes, degrees of freedom and *P* value noted<br>*Give P values as exact values whenever suitable.*
☒	☐	For Bayesian analysis, information on the choice of priors and Markov chain Monte Carlo settings
☒	☐	For hierarchical and complex designs, identification of the appropriate level for tests and full reporting of outcomes
☒	☐	Estimates of effect sizes (e.g. Cohen's *d*, Pearson's *r*), indicating how they were calculated

Our web collection on statistics for biologists contains articles on many of the points above.

Software and code

Policy information about availability of computer code

Data collection	Thermo Xcalibur (4.4)
Data analysis	Spectronaut (version 16), Perseus (1.6.7.0), GraphPad Prism (version 9), Image J (Fiji, 2.9.0), FlowJo (10.8.1), XDS (Version November 3, 2014), Phaser (version 2.5.6), COOT (version 0.8), Phenix (version 1.9-1692), ChymeraX (1.2.5), Octet Data Analysis HT software (Release 11.1)

For manuscripts utilizing custom algorithms or software that are central to the research but not yet described in published literature, software must be made available to editors and reviewers. We strongly encourage code deposition in a community repository (e.g. GitHub). See the Nature Portfolio guidelines for submitting code & software for further information.

Data

Policy information about availability of data

All manuscripts must include a data availability statement. This statement should provide the following information, where applicable:

- Accession codes, unique identifiers, or web links for publicly available datasets
- A description of any restrictions on data availability
- For clinical datasets or third party data, please ensure that the statement adheres to our policy

The crystal structure of N8C_Fab3b in complex with the neddylated CUL1 WHB domain has been deposited in the Protein Data Bank (PDB) under the accession code 8CAF. All mass spectrometry data have been deposited on the ProteomeXchange Consortium via the PRIDE database with the dataset identifier PXD039649. Expression plasmids for the N8C_Fab suite are available from Addgene (addgene.org). The PDB structure 1N8Z was used for molecular replacement to solve the

structure of N8C_Fab3b bound to NEDD8-CUL1(WHB). For structural comparison of N8C_Fab3b with NEDD8-CUL1(WHB) complex the PDB structures of 6TTU, 7B5L, and 7ONI were used.

Human research participants

Policy information about studies involving human research participants and Sex and Gender in Research.

Reporting on sex and gender	N/A
Population characteristics	N/A
Recruitment	N/A
Ethics oversight	N/A

Note that full information on the approval of the study protocol must also be provided in the manuscript.

Field-specific reporting

Please select the one below that is the best fit for your research. If you are not sure, read the appropriate sections before making your selection.

☒ Life sciences ☐ Behavioural & social sciences ☐ Ecological, evolutionary & environmental sciences

For a reference copy of the document with all sections, see nature.com/documents/nr-reporting-summary-flat.pdf

Life sciences study design

All studies must disclose on these points even when the disclosure is negative.

Sample size	No statistical analysis was carried out to predetermine sample size. Generally, we carried out at least three biological replicates for experiments involving cultured cells to ascertain result reproducibility.
Data exclusions	No data was excluded
Replication	All experiments were performed at least twice independent from each other with similar results.
Randomization	Male wild type C57BL/6N were randomly allocated into different groups for bone marrow derived macrophage isolation and activation. No pre-selection of mice, other than by sex and age as required by study design was performed.
Blinding	Blinding is not relevant to this study. All reported data are not subjective but based on quantitative determinations.

Reporting for specific materials, systems and methods

We require information from authors about some types of materials, experimental systems and methods used in many studies. Here, indicate whether each material, system or method listed is relevant to your study. If you are not sure if a list item applies to your research, read the appropriate section before selecting a response.

Materials & experimental systems

n/a	Involved in the study	
☐	☒	Antibodies
☐	☒	Eukaryotic cell lines
☒	☐	Palaeontology and archaeology
☐	☒	Animals and other organisms
☒	☐	Clinical data
☒	☐	Dual use research of concern

Methods

n/a	Involved in the study	
☒	☐	ChIP-seq
☐	☒	Flow cytometry
☒	☐	MRI-based neuroimaging

Antibodies

Antibodies used	N8C_Fab suite (this paper)

Antibodies used	anti-M13-HRP antibody (GE Healthcare #27-9421-01, 1:5000)<br>anti-Kappa-HRP (Southern Biotech #2060-05, 1:5000)<br>anti-CUL1 (Santa Cruz Biotechnology, #sc–17775, 1:1000)<br>anti-CUL2 (abcam, # ab166917, 1:1000)<br>anti-CUL3 (Bethyl Laboratories, #A301-109A, 1:1000)<br>anti-CUL4A (Bethyl Laboratories, #A300-739A, 1:1000)<br>anti-CUL5 (abcam, #ab184177, 1:1000)<br>anti-SKP1 (Cell Signaling Technology, #2156, 1:1000)<br>anti-BTRC (Cell Signaling Technology, #4394,1:1000)<br>anti-ELOC (Biolegend, #613101, 1:1000)<br>anti-DDB1 (abcam, # ab109027, 1:1000)<br>anti-CRBN (Sigma, #HPA045910, 1:1000)<br>anti-BRD4 (Cell Signaling Technology, #13440, 1:1000)<br>anti-GAPDH (Cell Signaling Technology, #2118, 1:5000)<br>goat anti-rabbit-HRP (#31460, ThermoFisher Scientific, 1:5000)<br>donkey anti-mouse-HRP (Jackson ImmunoResearch, #715-035-150, 1:5000)<br>Streptavidin-HRP (Cell Signaling Technology, #3999,1:5000)
Validation	N8C_Fab suite - validated in this paper (specific binding of neddylated CUL1,CUL2,CUL3,CUL4A, CUL4B over non-neddylated CUL1,CUL2,CUL3,CUL4A,CUL4B, CUL5, neddylated CUL5, and NEDD8; binding to human and mouse protein (tested) and other mammals (by similarity))<br>anti-M13-HRP antibody (GE Healthcare #27-9421-01, 1:5000) - (https://www.sigmaaldrich.com/DE/en/product/sigma/ge27942101)<br>anti-Kappa-HRP (Southern Biotech #2060-05, 1:5000) - (Reacts with human κ light chains, cross adsorption with Human λ light chains; may react with κ light chains from other species - https://www.southernbiotech.com/goat-anti-human-kappa-hrp-2060-05)<br>anti-CUL1 (Santa Cruz Biotechnology, #sc–17775, 1:1000) - (https://www.scbt.com/p/cul-1-antibody-d-5?productCanUrl=cul-1-antibody-d-5&_requestid=675840 - CUL-1 (D-5) is recommended for detection of CUL-1 of mouse, rat and human origin by Western Blotting)<br>anti-CUL2 (abcam, # ab166917, 1:1000) - (https://www.abcam.com/products/primary-antibodies/cullin-2cul-2-antibody-epr31042-ab166917.html; reacts with mouse, rat, human protein)<br>anti-CUL3 (Bethyl Laboratories, #A301-109A, 1:1000) - (https://www.fortislife.com/products/primary-antibodies/rabbit-anti-cul3-antibody/BETHYL-A301-109; binds human, and mouse protein)<br>anti-CUL4A (Bethyl Laboratories, #A300-739A, 1:1000) - (https://www.fortislife.com/products/primary-antibodies/rabbit-anti-cul4a-antibody/BETHYL-A300-739; binds human, and mouse protein)<br>anti-CUL5 (abcam, #ab184177, 1:1000) - (https://www.abcam.com/products/primary-antibodies/cullin-5cul-5-antibody-epr14725-ab184177.html; binds human, mouse, rat protein)<br>anti-SKP1 (Cell Signaling Technology, #2156, 1:1000) - (https://www.cellsignal.com/products/primary-antibodies/skp1-antibody/2156; Skp1 Antibody detects endogenous levels of total Skp1 protein of human, mouse, and rat origin)<br>anti-BTRC (Cell Signaling Technology, #4394,1:1000) - (https://www.cellsignal.com/products/primary-antibodies/b-trcp-d13f10-rabbit-mab/4394; β-TrCP (D13F10) Rabbit mAb detects endogenous levels of total β-TrCP protein of human, mouse, rat, and monkey origin. The antibody also recognizes a 40 kDa band of unknown origin.)<br>anti-ELOC (Biolegend, #613101, 1:1000) - (https://www.antibodypedia.com/gene/12329/ELOC/antibody/540261/613101)<br>anti-DDB1 (abcam, # ab109027, 1:1000) - (https://www.abcam.com/products/primary-antibodies/ddb1-antibody-epr6089-ab109027.html - binds human and mouse proteins)<br>anti-CRBN (Sigma, #HPA045910, 1:1000) - (https://www.atlasantibodies.com/products/antibodies/primary-antibodies/triple-a-polyclonals/crbn-antibody-hpa045910/; Genetic validation in WB by siRNA knockdown. Binds human, mouse, rat protein.)<br>anti-BRD4 (Cell Signaling Technology, #13440, 1:1000) - (https://www.cellsignal.com/products/primary-antibodies/brd4-e2a7x-rabbit-mab/13440; BRD4 (E2A7X) Rabbit mAb recognizes endogenous levels of total BRD4 protein. This antibody specifically recognizes the BRD4 long isoform (UniProt #O60885-1) and does not recognize other BRD4 isoforms.)<br>anti-GAPDH (Cell Signaling Technology, #2118, 1:5000) - (https://www.cellsignal.com/products/primary-antibodies/gapdh-14c10-rabbit-mab/2118, GAPDH (14C10) Rabbit mAb detects endogenous levels of total GAPDH protein of human, mouse, rat, monkey, bovine, and pig origin)<br>goat anti-rabbit-HRP (#31460, ThermoFisher Scientific, 1:5000) - (https://www.thermofisher.com/antibody/product/Goat-anti-Rabbit-IgG-H-L-Secondary-Antibody-Polyclonal/31460. Product # 31460 reacts with the heavy chains of rabbit IgG and with the light chains common to most rabbit immunoglobulins, but does not react against non-immunoglobulin serum proteins. However, this antibody may cross-react with immunoglobulins from other species and with SuperBlock® Blocking Buffers.)<br>donkey anti-mouse-HRP (Jackson ImmunoResearch, #715-035-150, 1:5000) - (https://www.jacksonimmuno.com/catalog/products/715-035-150; reacts with mouse IgG (H+L) with minimal cross reactivity with Bovine, Chicken, Goat, Guinea Pig, Syrian Hamster, Horse, Human, Rabbit, Sheep Serum Proteins)<br>Streptavidin-HRP (Cell Signaling Technology, #3999,1:5000) - (https://www.cellsignal.com/products/wb-ip-reagents/streptavidin-hrp/3999; Streptavidin has a remarkably high affinity for its natural ligand, biotin. The complex and irregular structure of the biotin-binding site makes it highly optimized for biotin binding and confers great specificity to the streptavidin-biotin complexes.)

Eukaryotic cell lines

Policy information about cell lines and Sex and Gender in Research

Cell line source(s)	293T (ACC 635), K562 (ACC 10), CAL-33 (ACC 447) and A549 cells (ACC 107) were obtained from Deutsche Sammlung von Mikroorganismen und Zellkulturen (DSMZ). Jurkat (TIB-152), SK-N-AS (CRL-2137), SK-OV-3 (HTB-77), Hep G2 (HB-8065), PC3 (CRL-1435) and SK-BR-3 cells, were purchased from American Type Culture Collection (ATCC). High Five™ Cells (BTI-TN-5B1-4) were purchased from ThermoFisher Scientific (Catalog number: B85502). Sf9 cells were purchased from ThermoFisher Scientific (Catalog number: 12659017)
Authentication	None of the cell lines used were authenticated.

Mycoplasma contamination	Cells were routinely tested for Mycoplasma using a biochemical test (Lonza's MycoAlert ® Mycoplasma Detection Assay) and tested negative.
Commonly misidentified lines (See ICLAC register)	No commonly misidentified lines were used in the study.

Animals and other research organisms

Policy information about studies involving animals; ARRIVE guidelines recommended for reporting animal research, and Sex and Gender in Research

Laboratory animals	8 to 10 week old male wild type C57BL/6N mice were used in this study.
Wild animals	No wild animals were used in this study.
Reporting on sex	Only male mice were used in this study.
Field-collected samples	No field-collected samples were used in this study.
Ethics oversight	Maintenance of mice at the animal facility of the Max Planck institute of Biochemistry under pathogen-free conditions and the use of mice for organ isolation was approved by the Government of Upper Bavaria.

Note that full information on the approval of the study protocol must also be provided in the manuscript.

Flow Cytometry

Plots

Confirm that:

☒ The axis labels state the marker and fluorochrome used (e.g. CD4-FITC).

☒ The axis scales are clearly visible. Include numbers along axes only for bottom left plot of group (a 'group' is an analysis of identical markers).

☒ All plots are contour plots with outliers or pseudocolor plots.

☒ A numerical value for number of cells or percentage (with statistics) is provided.

Methodology

Sample preparation	K562 cells were plated in 96-well plates and treated with indicated concentrations of MLN4924 for 2h. Cells were washed two times with PBS and then fixed with paraformaldehyde (ThermoFisher Scientific) for 10 min at room temperature, followed by cell permeabilization using ice cold methanol at -20C for 1 h and two washes with PBS-BSA (PBS containing 0.5% bovine serum albumin and 0.1% sodium azide). Cells were incubated with ~0.002 mg/ml N8C_Fab3b-Alexa FluorTM 647 in PBS-BSA for 1h at room temperature while shaking. Samples were washed twice with PBS-BSA and measured on an Attune NxT (ThermoFisher Scientific) flow cytometer. Mean fluorescent intensities (MFI) were extracted using FlowJo (BD Bioscience) and values normalized to the minimal and maximal MFI as averaged across replicates. Samples were plotted in Prism 9 (GraphPad) and the dose-response curve generated using the “sigmoidal dose-response” analysis function.
Instrument	Attune NxT (ThermoFisher Scientific)
Software	FlowJo 10.8.1
Cell population abundance	The population of single K562 cells based on FSC/SSC was ~50%.
Gating strategy	Single cells were picked based on FSC-H/SSC-A and subsequent SSC-A/SSC-H plots. A 637 nM laser with a 670/14 gate was used to measure AF647 fluorescence.

☒ Tick this box to confirm that a figure exemplifying the gating strategy is provided in the Supplementary Information.
