# Supplementary figures and images for: Activity-based profiling of cullin–RING E3 networks by conformation-specific probes
Source: Nat Chem Biol. 2023 Aug 31;19(12):1513–23. doi: 10.1038/s41589-023-01392-5 (PMC10667097; doi:10.1038/s41589-023-01392-5)

Figure 2

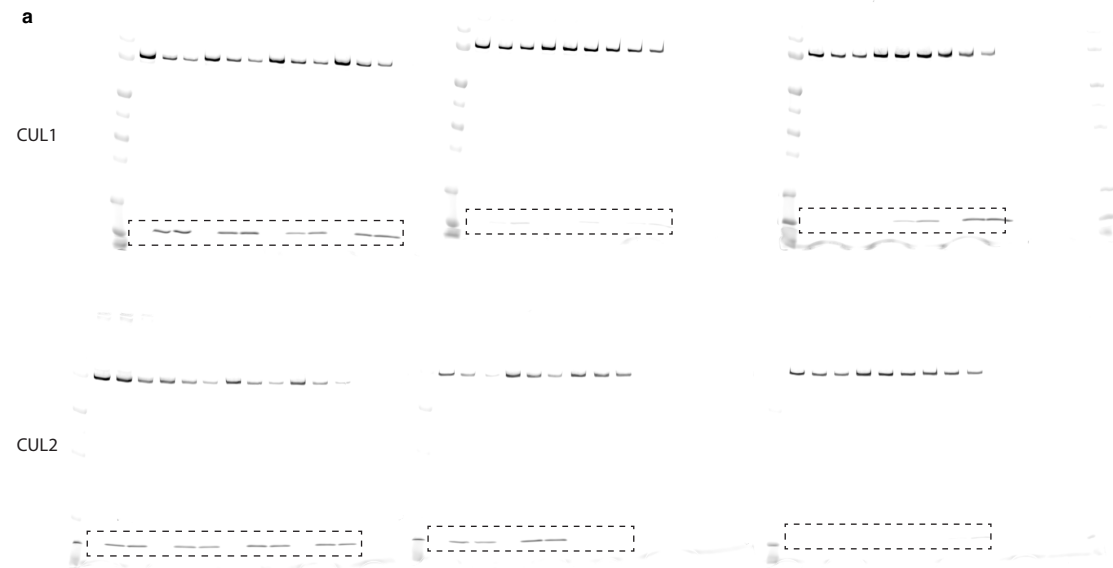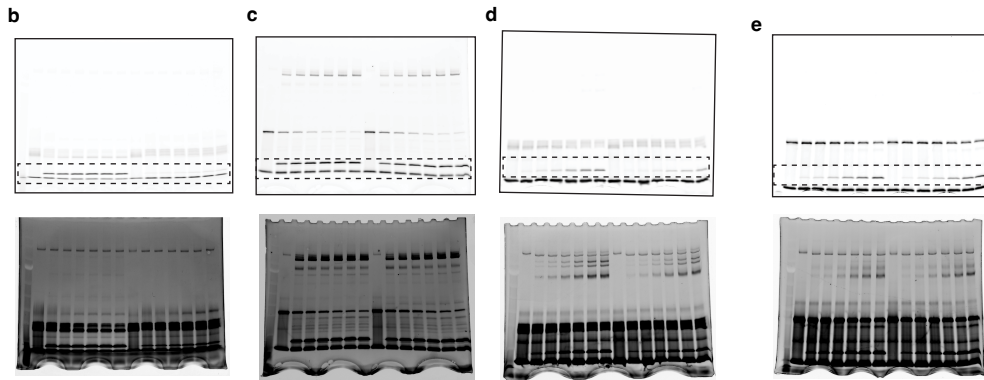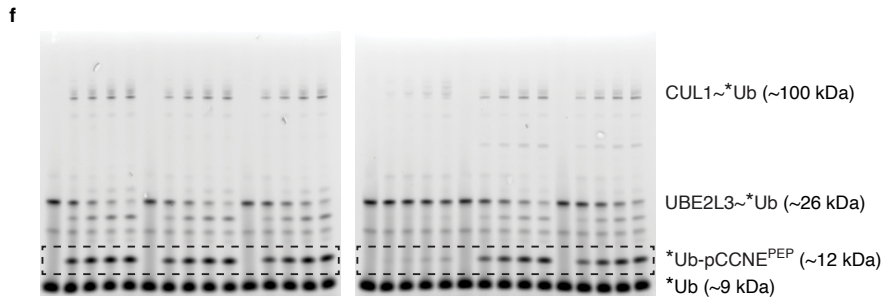

Supplement: Supplementary file 5 — Unprocessed gels. [file 41589_2023_1392_MOESM5_ESM.pdf]
