# Supplementary material for: Activity-based profiling of cullin–RING E3 networks by conformation-specific probes
Source: Nat Chem Biol. 2023 Aug 31;19(12):1513–23. doi: 10.1038/s41589-023-01392-5 (PMC10667097; doi:10.1038/s41589-023-01392-5)

CUL1#1

293T/A549/SK-N-AS/SKOV3

K562/Jurkat

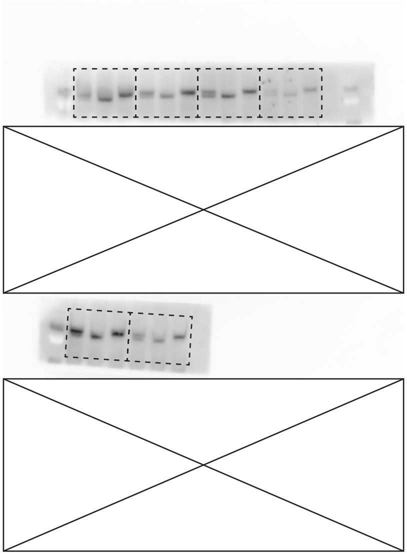

CUL4A#1

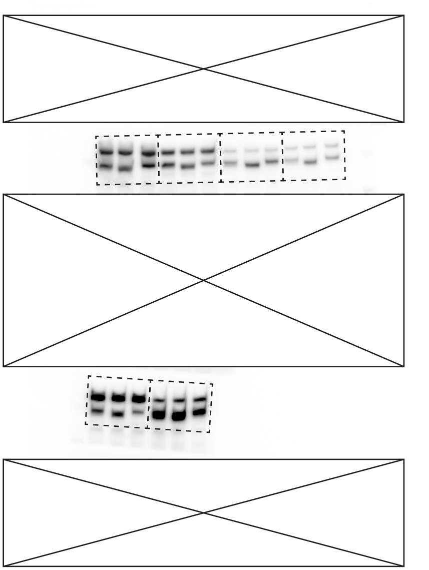

GAPDH#1

293T/A549/SK-N-AS/SKOV3

K562/Jurkat

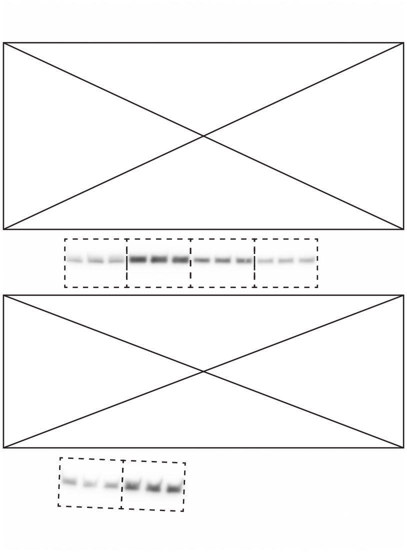

Marker

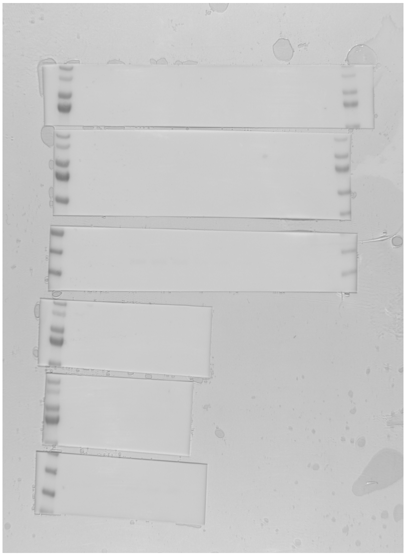

CUL1#1

CAL-33/PC-3/HepG2/SK-BR-3

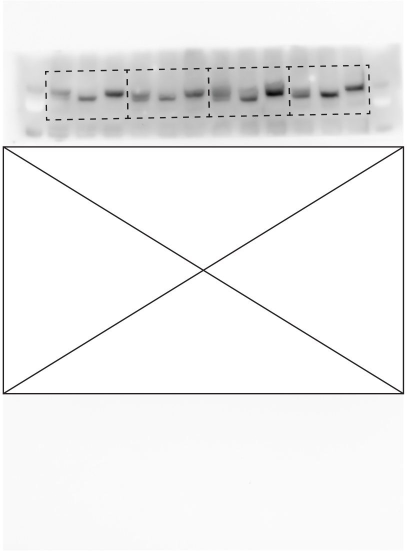

CUL4A#1

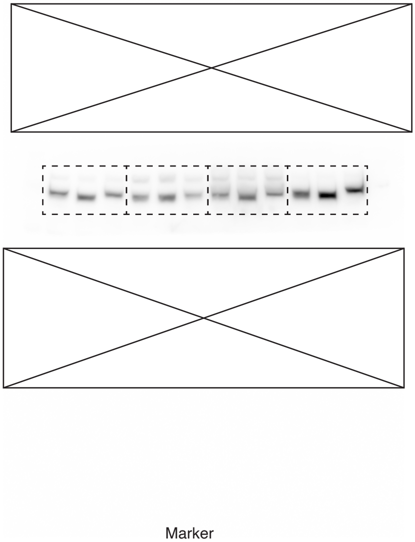

GAPDH#2

CAL-33/PC-3/HepG2/SK-BR-3

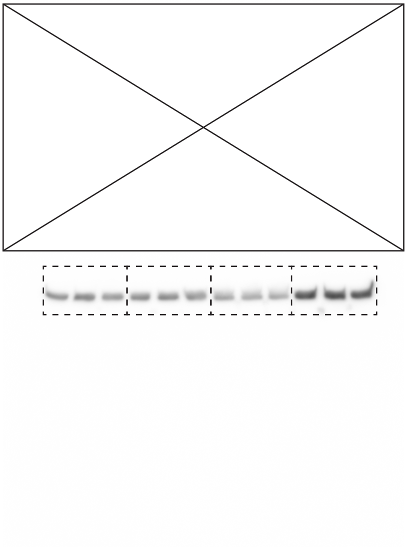

Marker

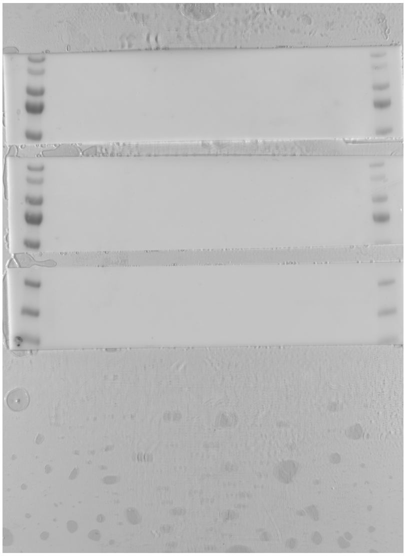

Supplement: Supplementary file 12 — Unprocessed western blots. [file 41589_2023_1392_MOESM12_ESM.pdf]
